# Review of therapeutic mechanisms and applications based on SARS-CoV-2 neutralizing antibodies

**DOI:** 10.3389/fmicb.2023.1122868

**Published:** 2023-03-16

**Authors:** Mingtao Liu, Hui Gan, Zhiman Liang, Li Liu, Qiwen Liu, Yiyin Mai, Huihuang Chen, Baoying Lei, Shangwei Yu, Huihui Chen, Peiyan Zheng, Baoqing Sun

**Affiliations:** ^1^National Center for Respiratory Medicine, The First Affiliated Hospital of Guangzhou Medical University, National Clinical Research Center for Respiratory Disease, State Key Laboratory of Respiratory Disease, Guangzhou Institute of Respiratory Health, Guangzhou, China; ^2^Guangzhou Medical University, Guangzhou, China

**Keywords:** SARS-CoV-2, COVID-19, neutralizing antibody, immunity, immunoassay

## Abstract

COVID-19 pandemic is a global public health emergency. Despite extensive research, there are still few effective treatment options available today. Neutralizing-antibody-based treatments offer a broad range of applications, including the prevention and treatment of acute infectious diseases. Hundreds of SARS-CoV-2 neutralizing antibody studies are currently underway around the world, with some already in clinical applications. The development of SARS-CoV-2 neutralizing antibody opens up a new therapeutic option for COVID-19. We intend to review our current knowledge about antibodies targeting various regions (i.e., RBD regions, non-RBD regions, host cell targets, and cross-neutralizing antibodies), as well as the current scientific evidence for neutralizing-antibody-based treatments based on convalescent plasma therapy, intravenous immunoglobulin, monoclonal antibodies, and recombinant drugs. The functional evaluation of antibodies (i.e., *in vitro* or *in vivo* assays) is also discussed. Finally, some current issues in the field of neutralizing-antibody-based therapies are highlighted.

## 1. Introduction

The global pandemic of coronavirus disease 2019 (COVID-19), caused by severe acute respiratory syndrome coronavirus 2 (SARS-CoV-2) wreaked economic and political havoc on the international community, posing grave hazards to human life (Cheng and Shan, 2020). The pathophysiology and therapy of SARS-CoV-2 have been the subject of research by scholars, government organizations, and private firms from all around the world. As a result of increased research, therapeutic antibodies and vaccines are emerging. At the time of writing, emerging immunotherapy primarily consists of interferon-based immunotherapy, antibody-based immunotherapy, management of the cytokine storm, and active immunotherapy – vaccine (Esmaeilzadeh and Elahi, 2021). Neutralizing antibody therapy is a mainstay among them. The term “neutralizing antibody therapy” refers to the competitive or non-competitive binding of antibodies to target cells in order to limit virus adhesion and infection. This review focuses on the mechanism of action of neutralizing antibodies directed against different sites. The various forms of immunological drugs have been outlined, along with their advantages and limitations. Additionally, the effectiveness of antibody verification is mentioned. Finally, we examine the difficulties inherent in neutralizing antibody therapy.

## 2. Neutralizing antibody therapy mechanism

After SARS-CoV-2 infects the human body, it triggers a cascade of immune responses in which B lymphocytes produce neutralizing antibodies that can bind competitively or non-competitively with the virus’s surface proteins, preventing the virus from recognizing the invasion of the hACE-2 receptor into the cell.

**Figure 1:**
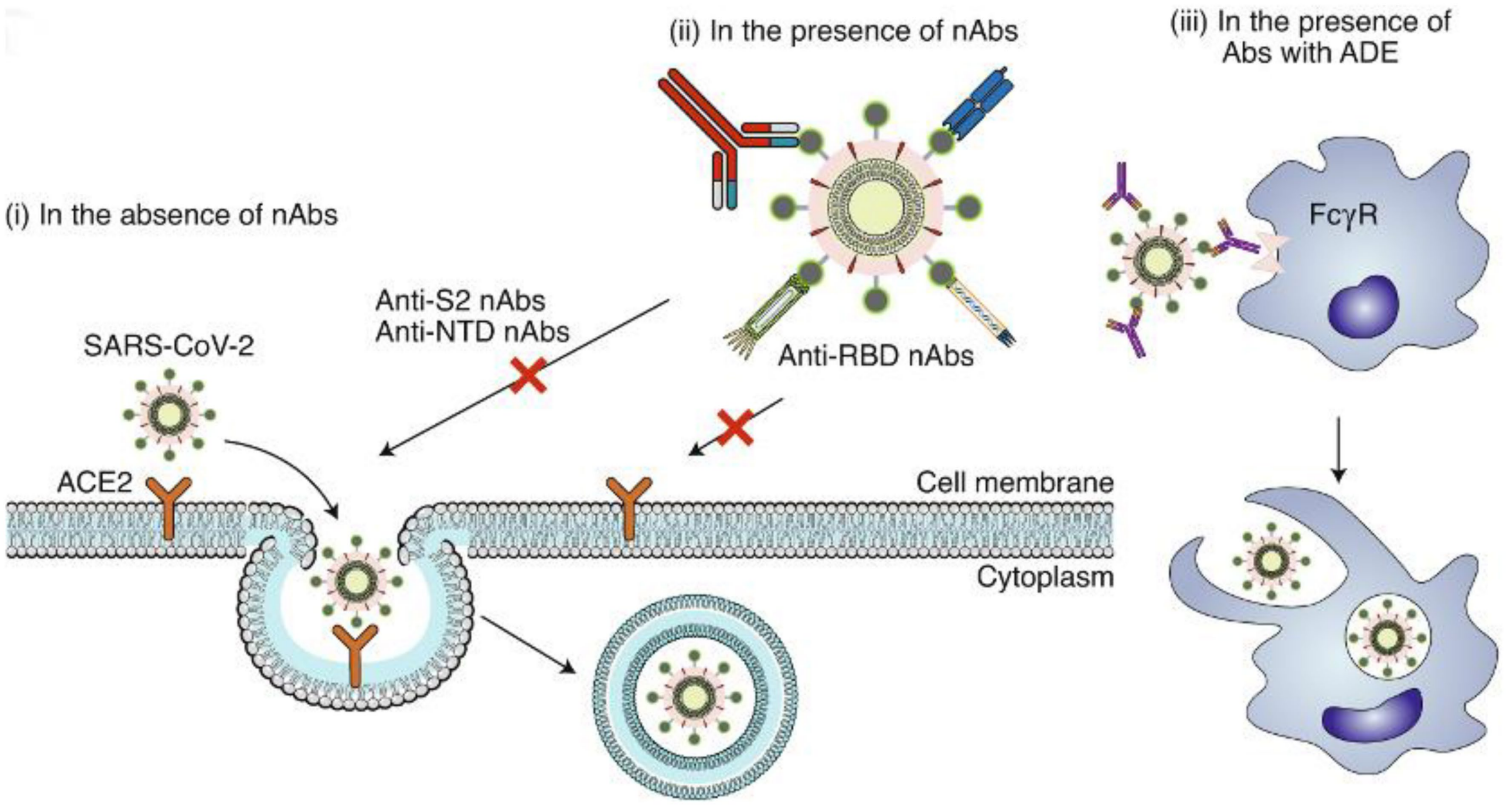
Potential mechanisms of action in SARS-CoV-2 neutralizing antibodies. (i) In the absence of nAbs, SARS-CoV-2 binds to the viral ACE2 receptor via the RBD, mediating viral entry into target cells. (ii) In the presence of RBD-specific nAbs, the antibodies bind to the RBD and inhibit RBD binding to ACE2, resulting in the inhibition of membrane fusion and the entry of the virus into the host cell. Some non-RBD-targeting nAbs may bind to the NTD, the S trimer or the S2 subunit (thus preventing conformational changes of S or inhibiting membrane fusion and viral entry). (iii) In the presence of nAbs with suboptimal or negligible neutralizing activity, the antibody-bound virions may enter cells (such as monocytes or macrophages) through the FcγR, leading to enhanced viral entry, viral replication or inflammation. Image and description courtesy of Jiang et al. (2020).

Human ACE2 protein (hACE2) is believed to be a critical target of the SARS-CoV S protein (Shirbhate et al., 2021). The virus initially attaches to gangliosides *via* the S protein, and subsequently to heparin sulfate (HS) and hACE2 *via* RBD recognition (Kombe Kombe et al., 2021). The virus then fuses with the host cell membrane *via* S2 and is internalized *via* endocytosis (Esmaeilzadeh and Elahi, 2021). Correspondingly, the potential mechanisms of action in SARS-CoV-2 neutralizing antibodies are shown in Figure 1.

### 2.1. Types of neutralizing antibody

**Figure 2:**
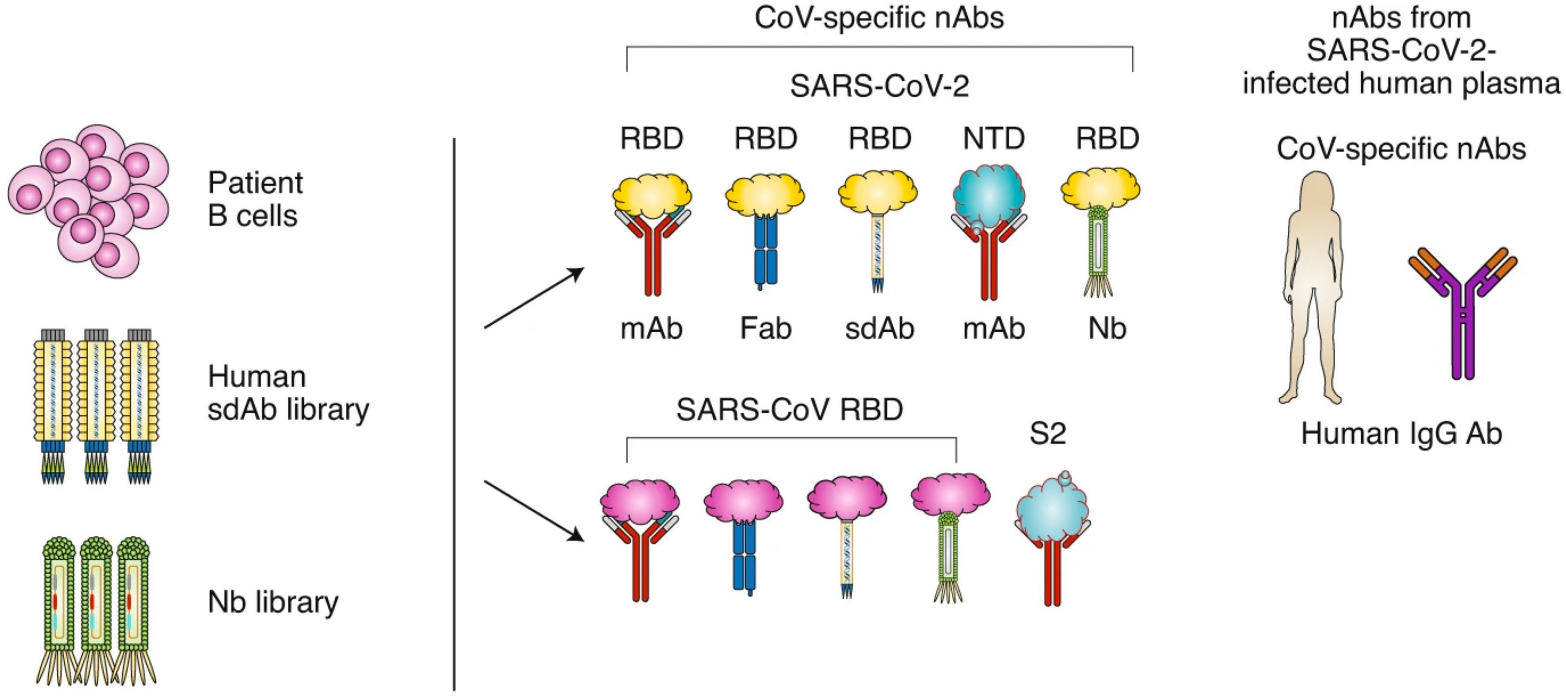
The generation type of SARS-CoV2 neutralizing antibodies. SARS-CoV-2 nAbs may be isolated from patients’ B cells, a library of human single-domain antibodies (sdAbs), or a library of nanobodies (Nbs). Different regions of the SARS-CoV-2 S protein are targeted by nAbs, including the RBD and NTD in the S1 subunit. SARS-CoV nAbs with cross-neutralization activity against SARS-CoV-2 may cross-react with the SARS-CoV-2 RBD or S2 subunit. Convalescent plasma from patients infected with SARS-CoV-2 could be used for the treatment of COVID-19. Image and description courtesy of Jiang et al. (2020).

Around 90% of individuals with mild to moderate SARS-CoV-2 infection develop anti-SARS-CoV-2 antibodies. The N and S proteins of SARS-CoV-2 are highly immunogenic, causing substantial amounts of IgA, IgM, and IgG to be produced by host cells. IgM and IgA are normally created after 7 days of infection, whereas IgG is produced between 10 and 18 days after infection. Titers of antibodies are stable for at least 5 months (Pisil et al., 2021). Although IgM is an early antibody that can contribute to and inhibit virus infection, its detection sensitivity may be lower than that of IgG and IgA, even during the early phases of virus infection (Long et al., 2020). IgA protects against viral infection by blocking the virus from attaching to the mucosa and is responsible for the majority of the early neutralizing antibody response to SARS-CoV-2 (Sterlin et al., 2021). The primary neutralizing antibody is IgG. They have high level of neutralizing activity and are involved in the humoral immunity process. Virus-specific IgG and IgM levels gradually increase during the course of a viral infection. In the third week following the onset of symptoms, IgM levels begin to fall, whereas IgG levels continue to climb to a stable state (Lagunas-Rangel and Chavez-Valencia, 2021). The generation type of SARS-CoV2 neutralizing antibodies was shown in Figure 2.

Each Ig molecule has a fragment antigen-binding (Fab) region and a fragment crystallizable (Fc) region that serve to detect antigens and mediate the effector functions of natural killer cells, macrophages, and the complement system, respectively. While IgM and IgA with the identical Fab domain have much stronger neutralizing ability than IgG monoclonal antibody, only IgG monoclonal antibody is now in clinical usage (Pisil et al., 2021)^.^ Clinical studies have demonstrated a large increase in IgM, IgA, and IgG against RBD from the first to the third week of the disease, as well as a significant increase in IgG antibodies against S1. Specific IgA levels are significantly greater in inpatient and intensive care patients with severe symptoms than in outpatient patients, and SARS-CoV-2 specific antibodies class switch to IgA earlier and more rapidly than IgG (Brynjolfsson et al., 2021).

Cheng et al. studied the humoral immune response of 357 vaccines over time, and concluded that NAb is positively correlated with IgG (Cheng et al., 2022). Similarly, Baral et al. (2021) analyzed the RBD-specific mAbs of 8 infected patients, and concluded that NAb competing with ACE2 may be a better predictor for virus-neutralizing antibody potency rather than binding affinity.

The correlation between anti-RBD antibody levels and NAbs remains unclear as there are contradictory reports on their association. Billon-Denis et al. (Chapman et al., 2021) studied two patients—one who presented with a strong anti-RBD IgG immune response that correlated with a low and rapidly waning NAb titre, whereas the other had strong IgG anti-RBD immune response, but high NAb titres. Hence, they propose that other host factors (e.g., age, gender, clinical severity) may be more dominant drivers of the immune response as opposed to NAb titres. In contrast, hence, blocking the interaction between RBD and ACE2 may be a useful surrogate for neutralization. The hindrance of the crystal structure of RBD-bound antibody inhibits viral binding to ACE2, thus blocking viral entry—suggesting that anti-RBD antibodies are mainly viral species-specific inhibitors. Another study also noted the correlation of NAb titres to anti-RBD IgG levels (Ho, 2020).

### 2.2. The mechanism of action of NAb

Numerous studies have highlighted the fact that viruses recognize and interact with host cells *via* the S glycoprotein, and that the coronavirus S glycoprotein is the primary target of post-infection neutralizing antibodies. The majority of SARS-CoV-2 neutralizing antibodies hinder SARS-CoV-2 S protein from binding to ACE2 on host cells (Klasse, 2014; Walls et al., 2020). Although 90% of the neutralizing antibodies specifically target RBD of the SARS-CoV-2 S1 protein (Piccoli et al., 2020), a few neutralizing antibodies target the NTD of S1 (Liu et al., 2021). While the S2 subunit of SARS-CoV-2 is considered a potential neutralizing target, several studies have discovered that S2 has no effect on ACE2 binding. Antibodies against the SARS-CoV-2 N protein are also produced in the human body, but their half-life is brief, and they have not been proved to neutralize the virus (Lagunas-Rangel and Chavez-Valencia, 2021). In recovered patients, the binding antibody against the nucleocapsid protein N had a greater titer than in non-recovered individuals. Because the N protein is contained within the virus and is not exposed to the surface of the virion, antibodies to the N protein have no neutralizing activity at all (Klasse and Moore, 2020). The following table summarizes the processes through which neutralizing antibodies target distinct antigen-binding domains.

Not only neutralizing antibodies as a definite treatment for patients with COVID-19, but a potential therapeutic for those with long-haul symptoms and central nervous system (CNS) deficits. In some cases, individuals who have recovered from COVID-19 or have been vaccinated may develop long-term symptoms, referred to as “long-haul” infections, which may include central nervous system deficits such as brain fog, fatigue, and difficulty concentrating, have been reported by a significant proportion of individuals who have recovered from COVID-19 (Korompoki et al., 2021; Mehandru and Merad, 2022; Shimohata, 2022). While the exact mechanisms underlying these long-term symptoms are unknown, several researchers suggest that they may be related to the persistence of viral particles in the body, ongoing inflammation, and/or changes in the immune system (Azkur et al., 2020; Tay et al., 2020; Dispinseri et al., 2021). The role of neutralizing antibodies in these cases is also not yet clear, but some studies have suggested that patients with higher levels of neutralizing antibodies may be less likely to experience long-term symptoms (Augustin et al., 2021; Rank et al., 2021). One study reported by Huang found that the SARS-CoV-2 infection mostly often appears with CNS syndrome and even last for weeks and months (Huang and Fishell, 2022). Then Hirzel discovered that patients with COVID-19 who developed neurological symptoms had lower levels of neutralizing antibodies compared to patients without neurological symptoms, which may suggest that neutralizing antibodies may play a role in protecting against CNS involvement in COVID-19 (Hirzel et al., 2022). The implications of neutralizing antibodies in these cases are not fully understood at this time. Research is ongoing to determine the relationship between neutralizing antibodies and long-haul COVID-19 symptoms and how they may be used to prevent or treat them.

#### 2.2.1. Neutralizing antibody against SARS-CoV-2 RBD

The RBD antigen domain of S1 is critical because it identifies and binds to the HACE-2 receptor, facilitating virus attachment to host cells. Neutralizing antibodies usually inhibit the virus in neutralizing antibody therapy by blocking RBD-ACE2 binding pathways. It has been demonstrated that the majority of neutralizing antibodies from the PMBC of convalescent patients compete with ACE2 for binding to RBD. At the moment, the most efficient antibodies are always directed against the ACE2-binding surface on the RBD of SARS-CoV-2 S protein (Zhou et al., 2021). Kim et al. showed that the neutralizing antibody CT-P59 causes complete steric hindrance by binding to the RBD of SARS-CoV-2 S protein, preventing the virus from binding to ACE2, and they found that CT-P59 can significantly inhibit the viral replication of clinical isolates, wild-type, and D614G variants by *in vitro via* the plaque reduction neutralization test (PRNT; Kim et al., 2021). Deyong et al. discovered that CA521FALA has three major advantages: direct competitive binding with ACE2, binding to all three RBDs of single spike simultaneously and bivalent binding of a single IgG. They speculated that CA521FALA IgG may use a mechanism similar to ACE2 to induce RBD transformation from a closed to an open configuration, allowing CA521FALA to bind more efficiently to RBD (Song et al., 2021).

The receptor-binding motif (RBM) is a small segment of the RBD structure, and the RBM overlaps the ACE2 binding site. Anti-RBM neutralizing antibodies can bind to the spike receptor binding domain RBM of SARS-CoV-2 competitively with ACE2, locking the RBD conformational state in the “down” conformational state. As a result, the virus is blocked from accessing the target receptor (Corti et al., 2021). This type of antibody comprises S2M11 antibody (Verkhivker and Di Paola, 2021), C144 (Corti et al., 2021), etc. P4A1 interacts directly with the bulk of the RBMs of the spike receptor binding domain, resulting in the steric hindrance of S protein binding to hACE2. This is a potent monoclonal antibody whose binding epitope almost entirely covers the majority of hACE2 binding sites. This wide coverage may allow the antibody efficiently attack spike mutants, making it a good treatment option for immunocompromised and susceptible populations (Guo et al., 2021). Additionally, the neutralizing antibody S230 against SARS-CoV RBM can function as a receptor mimicry prior to the virus interacting with host cells, causing conformational changes in the fused S protein that inactivate it and prevent viral infection (Walls et al., 2019). According to some researchers, the neutralizing antibody against SARS-CoV-2 RBM may function this mechanism (Corti et al., 2021). Some non-RBM monoclonal antibodies have been shown to block viral infection by blocking structural rearrangement of S protein and spatially interfering with ACE2 participation (Corti et al., 2021).

#### 2.2.2. Neutralizing antibodies against SARS-CoV-2 NTD

Although most neutralizing antibodies target the RBD region of the S protein, some researchers have shown that several SARS-CoV-2 neutralizing antibodies target susceptible epitopes in non-RBD regions. The N-terminal domain NTD of the S1 subunit is considered as a substitute and/or complementary target for neutralizing antibodies. Lok detailed the mechanism of neutralization anti-NTD super antibodies, one of which was that anti-NTD super antibodies were unable to inhibit S protein from binding to the ACE2 receptor (Lok, 2021). Makdasi et al. discovered that none of the anti-NTD monoclonal antibodies they isolated was neutralizing activity (Makdasi et al., 2021). The therapeutic efficacy of anti-NTD monoclonal antibodies remains unknown, and proper clinical trials are required to prove their efficacy. Simultaneously, surprising evidence demonstrated that while the binding epitope region of the SARS-CoV-2 NTD neutralizing antibody did not overlap with or compete with ACE2 binding, certain antibodies, such as 4A8mAb (Imai et al., 2020), may also have substantial neutralizing activity. Certain neutralizing antibodies can selectively bind to S1-NTD, preventing it from interacting with the host cell. According to MD simulations, small molecules targeting S1-NTD (Di Gaetano et al., 2021) can disrupt RBD and ACE-2 interactions. Certain investigations hypothesized that some anti-NTD antibodies could neutralize SARS-CoV-2 by blocking the conformational change of S protein (Imai et al., 2020). Through crystal structural research, it was discovered that the NTD antibody binding S protein caused RBD to be in a “down” state, preventing RBD from binding to ACE2 (Jin et al., 2021). According to John et al., NTD of the S1 subunit reduces S protein binding to cell surface receptors *via* NTD-GBD-binding inhibition, and prevent the formation of the S-ACE2 complex (Kombe Kombe et al., 2021). Jin et al. found that by combining NTD neutralizing antibodies against SARS-CoV-2 S1 with RBD neutralizing antibodies against SARS-CoV-2 S1, antiviral efficacy could be enhanced (Jin et al., 2021).

#### 2.2.3. Antiandrogen drugs inhibit SARS-CoV-2 entry

TMPRSS2 is a widely recognized androgen-regulated gene associated with prostate cancer (Afar et al., 2001). The infiltration of the coronavirus into the cell through the viral spike protein (S protein) binding precision to a host cell receptor and human angiotensin-converting enzyme 2 (ACE2), which is made possible through the calculated actions of the serine protease TMPRSS2, priming the way for the virus to gain entry and wreak its destructive path (Min and Sun, 2021). TMPRSS2 promotes SARS-CoV-2 entry by two separate mechanisms: ACE2 interaction/cleavage, which might promote viral uptake, and SARS-CoV-2-S cleavage, which activates the S protein for membrane fusion (Hoffmann et al., 2020). It implicated that male bias in COVID-19 severity and mortality, which provides a strong rationale for androgen deprivation or anti-androgen therapy in men with SARS-CoV-2 infection. Deng et al. demonstrated that the expression of SARS-CoV-2 host cell receptor ACE2 and co-receptor TMPRSS2 is regulated in part by the male sex hormone androgen, and the cell entry of SARS-CoV-2 spike pseudovirus can be blocked by androgen deprivation, anti-androgens, or clinically proven inhibitors of TMPRSS2 such as camostat (Deng et al., 2021). Hoffmann and his colleagues’ research also revealed that clinically-proven protease inhibitor camostat mesylate inhibits SARS-CoV-2 infection by blocking the virus-activating host cell protease TMPRSS2 (Hoffmann et al., 2021). In preclinical research, the TMPRSS2 inhibitor nafamostat also reduced SARS-CoV-2 pulmonary infection in a mouse model of COVID-19 (Li et al., 2021). Chen et al. identified FDA-approved small molecules that reduce surface expression of TMPRSS2 that can limit SARS-CoV-2 entry in both live and pseudoviral *in vitro* models (Chen et al., 2021a). McCoy and his colleagues demonstrated that antiandrogens drug proxalutamide treatment reduced the hospitalization rate by 91% compared to usual care (McCoy et al., 2021). However, the newly emerged variants Omicron inefficiently uses the cellular protease TMPRSS2 for cell entry, and deletion of TMPRSS2 affected Omicron entry to a less extent than wild-type SARS-CoV-2 (HKU-001a) and previous variants (Meng et al., 2022; Zhao et al., 2022). It indicates that antibodies generated against TMPRSS2 may be less effective in Omicron variant. In a randomized clinical trial of reduced expression of TMPRSS2 by antiandrogen therapies - degarelix did not result in amelioration of COVID-19 severity (Nickols et al., 2022). Therefore, more research is needed to confirm the effectiveness of antiandrogens drugs in treating COVID-19.

#### 2.2.4. Neutralizing antibody treatments with endogenous ACE2 ligands

The emergence of SARS-CoV-2, the virus responsible for the ongoing COVID-19 pandemic, has brought with it a renewed sense of urgency to develop effective treatments and vaccines (Mistry et al., 2022). One promising approach is the use of neutralizing antibodies to target the spike protein of the virus, which allows it to enter human cells. However, as we strive to combat this virus, it is essential that we also consider the potential impact of these treatments on the human body (Pantaleo et al., 2022). One area of concern is the interaction of neutralizing antibodies with ACE2, an endogenous protein present on the surface of many human cells. ACE2 plays a crucial role in regulating blood pressure and other physiological processes, and the spike protein of SARS-CoV-2 has a similar structure to ACE2, making it a potential target for neutralizing antibodies (Alfaleh et al., 2022; Eslami et al., 2022; Suvarnapathaki et al., 2022). Nevertheless, several potential ramifications of this interaction are still unresolved. For example, binding of neutralizing antibodies to membrane-associated ACE2 may prevent the protein from performing its normal functions, leading to changes in blood pressure or other physiological processes (Pizzato et al., 2022). Additionally, neutralizing antibodies may bind to soluble ACE2 in the blood, leading to decreased levels of the protein and exacerbating the effects of ACE2 deficiency (Ning et al., 2022). While the development of neutralizing antibody treatments is a vital step in the fight against COVID-19, it is essential that we also consider the potential impact of these treatments on the human body. Clinical studies are ongoing, and early data suggest that neutralizing antibody therapies may be associated with an increased risk of thrombotic events. However, it is important to keep in mind that the benefits of these treatments, such as preventing severe illness and death from COVID-19, outweigh the potential risks (Iba and Levy, 2022; Levi and van Es, 2022; Silva-Beltrán et al., 2022). Thus, our scientific community must continue to monitor the safety of these treatments and conduct further research to fully understand their potential impact on ACE2 and other physiological processes.

To our knowledge, one of the ways SARS-CoV-2 invades human cells is through a process known as endocytosis. This intricate mechanism involves the virus binding to receptors on the surface of host cells, such as the ACE2 receptor, and then being engulfed by the cell, thus allowing it to evade the body’s immune system and replicate within the host (Matveeva et al., 2022). However, in the ongoing battle against COVID-19, neutralizing antibody (nAb) therapy has emerged as a promising strategy to combat the virus. The goal of this therapy is to prevent the virus from binding to the host cell’s receptors by providing antibodies that bind to the virus and prevent it from entering the host cell (Kumari et al., 2022). But this virus is not one to be underestimated. It has been observed that the virus can evade nAb therapy by entering the cell through alternative endocytic pathways (Sanna et al., 2022; Zolfaghari et al., 2022). This highlights the need for continued research and development of new and innovative therapies to combat this resilient virus.

### 2.3. Cross-neutralizing antibodies

Numerous reports have surfaced regarding antibodies that cross-neutralize SARS-CoV-2 and other coronaviruses. The following sections provide an overview of cross-neutralizing antibodies directed against SARS-CoV-2, SARS-CoV, and seasonal human coronaviruses (HCoV).

Wu et al. revealed that certain SARS-CoV-2-induced NAB could cross-bind but not cross-neutralize SARS-CoV, i.e., could not prevent SARS-CoV infection, due to epitope or immunogenicity discrepancies between SARS-CoV-2 and SARS-CoV. The plasma of SARS-CoV-2 patients binds to SARS-CoV S protein but not to SARS-CoV-2 S protein (Dispinseri et al., 2020). Other researches have produced results that contradict the aforementioned. Zhang et al. discovered that not only did the individual serum produce a robust neutralizing antibody response to SARS-CoV-2 during the recovery period, but that some antibodies in the serum may cross-bind to SARS-CoV and MERS-CoV, resulting in neutralizing activity. The study exploited the varying sequence homology of the spike proteins of the three coronaviruses to explain why SARS-CoV exhibited a higher level of cross-neutralization than MERS-CoV in the sera of patients (Zhang et al., 2021). Certain neutralizing monoclonal antibodies (mAbs) specific for SARS-CoV have been shown to efficiently cross-neutralize SARS-CoV-2 by targeting its conserved epitope (Kombe Kombe et al., 2021). At the same time, some studies have discovered that the large C-terminus residues variation between SARS-CoV-2 and SARS-CoV has a considerable effect on neutralizing antibody cross-reactivity (Tian et al., 2020).

SARS-CoV and SARS-CoV-2 viruses are both members of the Coronavirus family and belong to the same genus (Adenoviridae). SARS-CoV-2 shares 79.6% sequence identity to SARS-CoV (Zhou P. et al., 2020), the N protein shares 90.52% homology with SARS-CoV (Xu et al., 2020), the S1 subunit shares 64% homology with SARS-CoV, and the S2 subunit shares 90% homology with SARS-CoV (Jaimes et al., 2020). Both virus may utilize ACE2 as a receptor for their host cell (Zhou P. et al., 2020). There are antibodies that cross-react with SARS-CoV and SARS-CoV-2, although the majority of these antibodies target the S2 subunit (Ramasamy et al., 2021) and N protein (Okba et al., 2020) which are conserved on S protein and have not been demonstrated to have neutralizing activity. The few cross-reactive antibodies with neutralizing activity are primarily directed towards RBD epitopes, preventing the virus from binding to ACE2 and triggering S1 dissociation (Wec et al., 2020), although the majority of them have a low affinity for viral antigens. EY6A and CR3022, for example, can cross-react with SARS-CoV-2 S1, but their affinity is low (Zhou D. et al., 2020).

Prior to the COVID-19 pandemic, a large number of people were exposed to seasonal human coronavirus and developed antibodies against them (Anderson et al., 2021). Seasonal human coronavirus (HCoV) infections, including those caused by HCoV-229E, -OC43, -NL63, and -HKU1, typically elicit cold symptoms in immunocompetent individuals (Hasoksuz et al., 2020). Khalid et al. investigated the connection between antibodies against HCoV-NL63 and 229E (α-HCoV), HKU1 and OC43 (β-HCoV), and SARS-CoV-2 cross-reactions. The results demonstrated that anti-SARS-CoV-2 S1 and S1-RBD antibodies had a high degree of cross-reactivity with anti-HKU1 SRBD and OC43 S1 antibodies, indicating that HCoV-HUK1 and HCoV-OC43 antigens were similar to SARS-CoV-2 antigens. They have the ability to induce SARS-CoV-2 to produce antibodies against S protein and cross-react with it. The link between the NL63 antibody and the 229E antibody was poor. Cross-reactive antibodies with neutralizing activity have also been reported to be related with antibodies on SARS-CoV-2 S1 and S1-RBD in studies using SARS-CoV-2 pseudovirus neutralization testing (Shrwani et al., 2021). Furthermore, they discovered that the degree of antibodies against SARS-CoV-2 and seasonal human coronavirus cross-reaction was inversely associated to age, which may partly explain why children are better able to resist severe COVID-19 disease than the elderly. Brodin et al. have also indicated that children are less likely to experience severe symptoms of COVID-19 compared to the elderly (Brodin, 2021). According to the Centers for Disease Control and Prevention (CDC), children under the age of 18 account for a small proportion of COVID-19 cases and hospitalizations, while children are less likely to require hospitalization or die from the disease, which might attribute to a strong innate immunity and more resistant to serious disease outcomes (Adams et al., 2020; Dhochak et al., 2020; Steinman et al., 2020). Moreover, the World Health Organization (WHO) states that people aged 60 years and above are at greater risk of developing severe illness or dying from COVID-19 (Organization WH, 2021). This is due to a range of factors, including age-related changes in the immune system and underlying health conditions (Bartleson et al., 2021). However, it is also crucial to note that this does not mean children are immune to the virus, and it is still important to take necessary precautions to protect them, as well as the most vulnerable members of society.

## 3. Immune drugs

Apart from convalescent plasma (CP) and intravenous immunoglobulin (IVIG), it is now possible to obtain synthetic monoclonal antibodies (mAbs). At the moment, the most recent discovery is a technique for manufacturing recombinant nano antibodies, which offers up a new path for the creation of therapeutic antibodies. Table 1 compares different immune drugs.

**Table 1:** Comparison of different immune drugs.

Immune drugs	Source	Preparation methods	Experiment effect	Advantages and disadvantages	Applicable timing	Half-lives	Ref.
Convalescent plasma (CP)	Patients with COVID-19 recovered	The blood center collects and prepares whole blood and plasma.	1. Prevented severe COVID-19, reduced pulmonary viral replication, and restricted pulmonary pathology changes in a hamster model.	Advantages:	Severe COVID-19 infection or hospitalized patient	No data.	Abuzakouk et al. (2021), Gharbharan et al. (2021), Haagmans et al. (2021), Terpos et al. (2021), Wendel et al. (2021), Yokoyama et al. (2021), Yonemura et al. (2021)
			2. Clinical effects:	The most easily accessible source of neutralizing antibodies against SARS-CoV-2.			
			i. Infusions were well tolerated, patients had improved, five patients experienced mild to moderate transfusion reactions.	Disadvantages:			
			ii. NAbs lacked long-term persistence in CP. CP antibodies do not improve the clinical status of critically ill and hospitalized patients.	Insufficient plasma donors，differences in the levels of nAbs in convalescent, antibody immune maintenance level, immune deficit, impact of plasma component and blood coagulation factor, new virus variant, poor response to treatment			
Intravenous immunoglobulin (IVIG)	Human or animal plasma	Commercial product	Contradictory clinical effects:	Advantages: Anti-infection, anti-viral replication, anti-inflammation, regulation of the complement cascade of SARS-CoV-2 activation, and regulation of autoimmunity	critically ill patients with COVID-19 within 48 h of ICU	18–32 day	Cao et al. (2021), Chen X. et al. (2021), Esmaeilzadeh et al. (2021), Farcet et al. (2021), Hou et al. (2021), Huang et al. (2021), Perricone et al. (2021), Xiang et al. (2021)
			1. It is often used for the combined treatment of SARS-CoV-2 complicated with other immune diseases. SARS-CoV-2 has a certain curative effect in critically ill patients. High-dose IVIG has been shown to be effective in patients with early and non-comorbidities.	Disadvantages: high cost, low potency, impurity problems, insufficient supply batches, and batch variances, ADE, transfusion-associated acute lung injury (TRALI), thrombus formation.			
			2. IVIG may increase the risk of viral clearance. IVIG adjuvant therapy did not improve in-hospital mortality or mechanical ventilation requirements.				
Monoclonal antibodies (mAbs)	1. COVID-19 convalescent patients’ plasma	1. Plasma: use single cell analysis to separate B cells and enrich B cells to extract monoclonal antibodies	1. Many mAbs have been shown to be effective in animal models.	Advantages:	REGN-COV2:post-exposure prophylaxis (prevention)；Bamlanivimab/etesevimab:high-risk ambulatory patients； Sotrovimab:hospitalized patient；Tixagevimab and Cilgavimab (Evusheld):pre-exposure prophylaxis of COVID-19.	No data.	Andreano et al. (2021), Baral et al. (2021), Brouwer et al. (2020), Cao et al. (2020), Chapman et al. (2021), Chen et al. (2021), Corti et al. (2021), Dong et al. (2020), Ju et al. (2020), Kim et al. (2021), Rogers et al. (2020), Tabll et al. (2021), Vaisman-Mentesh et al. (2020), Wang et al. (2020)
	2. Animal testing	2. Animal: inoculate the SARS-CoV-2 vaccine in animals and separate B cells to obtain monoclonal antibodies.	2. TY027, BRII-196, BRII-198 and SCTA01 are in Phase III clinical trials.	1. Neutralize SARS-CoV-2 and prevent virus infection			
	3. Hybridoma technique	3. Hybridoma technique	3. LY- COV555, REGN-COV2, Evusheld and CT-P59 have obtained EUA.	2. Bind only a specific cluster of antigens and can be precisely designed and optimized			
		4. *In vitro* culture method	4. REGN-COV2 was effective for the hemodialysis patients	3. Target the receptor-binding domain of the SARS-CoV-2 spike pro tein, thereby preventing viral entry into human cells through the angiotensin-converting enzyme 2 (ACE2) receptor			
			5. Bamlanivimab/etesevimab reduced the sars-cov-2 viral load in high-risk patients	Disadvantages:			
			6. Tixagevimab and cilgavimab (evusheld) were used to conduct the phase III tacp test for outpatient participants, and the results showed high-level results	1. Effect is affected by virus variation			
				2. It is easy to cause adverse drug reactions			
				3. Probably exist ADE			
				4. Large individual differences and high costs			
				5. Virus mutation escape antibody neutralization			
Nanobodies	1. Camelidae	PBMC were isolated after immunizing VHH transgenic mice. VHH gene was amplified and cloned into the phagocyte vector. After expression, enriched antibodies were screened by deep sequencing.	*In vitro* SARS-CoV-2 Spike pseudovirus neutralization test: the tandem trimer form of nanobodies effectively neutralized the virus and prevented escape.	Advantages:	Pre-clinical research.	No data.	Atagenix (2021), Jovcevska and Muyldermans (2020), Perween et al. (2021), Xue et al. (2016), Xu et al. (2021), Voss (2021)
	2. VHH transgenic mice			1. Genetic engineering operations can be carried out.			
	3. Phage display antibody library			2. It is small in size and can bind to relatively concealed sites.			
				3. Chemical is easy to prepare.			
				4. Stable physical and chemical properties, heat resistance, and tolerance to extreme pH.			

### 3.1. Convalescent plasma

Convalescent plasma (CP), an antibody-rich plasma from diseased individuals, is the most readily accessible source of neutralizing antibodies against SARS-CoV-2 and a passive immunotherapy option for infected and susceptible populations. The 1918 influenza pandemic demonstrated the effectiveness use of CP. This therapy has been successfully used to treat emerging infectious diseases (EID) such as severe acute respiratory syndrome (SARS), Middle East respiratory syndrome (MERS), and Ebola virus disease (Yonemura et al., 2021).

Convalescent plasma can suppress viral replication by neutralizing antibodies and reverse the inflammatory response *via* additional immunomodulatory mechanisms such as the complement system, antibody-dependent cellular toxicity, and phagocytosis (Esmaeilzadeh and Elahi, 2021). Additionally, it contributes to the suppression of SARS-CoV-2 shedding and accelerates viral and infected cell clearance. Clinical trials by Haagmans et al. demonstrated in a hamster model that using monoclonal antibodies or convalescent plasma prevented severe COVID-19 infection, weight loss, decreased pulmonary viral replication, and restricted pulmonary pathology changes. Plasma therapy efficacy is dependent on a high level of neutralizing antibody titer, and a decrease in neutralizing antibody titer results in a loss of protective effect (Haagmans et al., 2021). Many studies have shown the safety and efficacy of convalescent plasma in clinical practice. De Giorgi et al. studied 228 blood donors, finding that 97% had anti-SARS-CoV-2 antibodies at initial presentation, and 63% of 116 donors in follow-up analyses had detectable neutralizing antibody titers 11 months after recovery (De Giorgi et al., 2021). In this study, 102 patients received COVID-19 Convalescent Plasma (CCP), and the infusions were well tolerated. By the fifth day, 19 (18.6%) patients had improved, and 45.1% of the patients had improved by the fourteenth day. Five (4.8%) patients experienced mild to moderate transfusion reactions (Yokoyama et al., 2021).

Convalescent plasma gained traction as a therapy for SARS-CoV-2 infections in the early days of the pandemic. The concept was that neutralizing antibodies in the plasma of individuals who have recovered from COVID-19 could be passively transferred to newly-infected individuals to reduce their viral load and alter the course of the infection towards clinical improvement and recovery. Initial retrospective cohort studies suggested benefit with the infusion of high-titer convalescent plasma particularly when given early in the hospital course. Subsequently, data from randomized controlled trials emerged as the pandemic progressed that muddled the support for its use as a therapy for those infected with SARS-CoV-2. Indeed, the meta-analysis by Jorda et al. finds that convalescent plasma had no benefit as a therapy in COVID-19. This conclusion was also reached by the NIH and IDSA (Infectious Disease Society of America) who do not recommend giving convalescent plasma to hospitalized COVID-19 patients. However, the US FDA currently continues to allow for the use of high titer convalescent plasma under emergency useauthorization (EUA) for immunocompromised patients early in the course for COVID-19, even in the outpatient setting. Wirzet al. provides insight to the benefits of early use of convalescent plasma into patients infected with SARS-CoV-2; Only patients transfused before seroconversion, which *de facto* equates to early in the disease course, had demonstratable increase in plasma anti-SARS-CoV-2 antibody levels. Additionally, Yue et al. found the neutralizing ability of convalescent plasma is attenuated if collected prior to the emergence of variants of concern and is another consideration when choosing this treatment modality (Razonable and Chen, 2022).

### 3.2. Intravenous immunoglobulin

Intravenous immunoglobulin (IVIG) preparations are polyvalent antibodies derived from human or animal plasma with an 18-32-day half-life. IVIG is composed of normal IgG immunoglobulins seen in healthy blood donors. The bulk of immunoglobulin preparations are IgG monomers (> 96%), whereas some may contain trace levels of IgG dimers, Ig M, and Ig A. While trace soluble molecules such as human leukocyte antigens (HLA) and cytokines may be present, they do not contain immune complexes or contaminants with a large molecular weight. IVIG is frequently used to treat inflammatory diseases, immunodeficiency, and severe infections. It is particularly effective for patients with immunodeficiency and a suboptimal immune system response. People with primary and secondary immunodeficiencies (PID/SID) may benefit IVIG therapy. The therapy has been successfully employed in SARS and MERS clinics with favorable curative results (Esmaeilzadeh et al., 2021; Farcet et al., 2021).

Anti-infection, anti-viral replication, anti-inflammation, regulation of the complement cascade of SARS-CoV-2 activation, and regulation of autoimmunity can all be achieved with IVIG therapy. Number of studies have shown that IVIG is more successful in treating SARS-CoV-2. Xiang et al. found that IVIG was clinical effective in critically ill patients with COVID-19 and that initiating IVIG within 48 h of ICU significantly decreases mortality, mechanical ventilation use, and duration stay. Its efficacy may be connected to the severity of COVID-19 disease, with early IVIG use being associated with a shorter hospital stay and later use being associated with a longer hospital stay (Xiang et al., 2021). Cao et al. discovered that larger IVIG dosages were associated with a more rapid resolution of inflammatory and improved clinical outcomes in patients. High doses of IVIG were associated with a decreased 28-day mortality rate in patients who developed severe symptoms within 14 days, and the impact was more pronounced in those who got treatment early or did not have comorbidities (Cao et al., 2021). Between March and August 2020, no SARS-CoV-2 neutralizing antibody was detected in the IVIG batch. As the month advanced, the number of batches positive for SARS-CoV-2 neutralizing antibodies increases. Future vaccine-induced antibodies and antibodies against novel viral types will aid in improving IVIG efficiency (Farcet et al., 2021).

### 3.3. Monoclonal antibody

Monoclonal antibodies are highly homogenous antibodies created by a single B cell that binds only to a specific antigen cluster. Monoclonal antibodies are more successful than plasma treatment in convalescent patients because they can be precisely designed and refined, conveniently manipulated, and quality-controlled. The Fab segment of the monoclonal antibody can recognize the binding target antigen and interfere with the antigen’s biological function. In addition, the Fc segment can also connect to immune cells that express Fc receptors, activate the complement in the blood, and subsequently mediate immunological responses such as ADCC, ADCP, and CDC, and others.

Many viral infections, such as HIV-1 (Astronomo et al., 2021), respiratory synchro virus (RSV; Andabaka et al., 2013), and Ebola virus (Levine, 2019), have been successfully treated with monoclonal antibodies. There is no monoclonal antibody vaccine against SARS-CoV-2 on the market at the moment, and the monoclonal vaccine is still in development and not in use for COVID-19. The monoclonal neutralizing antibodies are mostly directed against the RBD of SARS-CoV-2 S protein. Because the S protein RBD serve as host cell-binding site, and neutralizing monoclonal antibodies against RBD have been shown to prevent viral infection, it is a prime target for neutralizing antibody development (Ho, 2020).

Numerous research groups have isolated monoclonal neutralizing antibodies against SARS-CoV-2 over the last 2 years, the majority of which were prepared by isolating plasma from COVID-19 convalescent patients (Brouwer et al., 2020; Ju et al., 2020; Andreano et al., 2021), with a few obtained through animal testing (Dong et al., 2020; Chen et al., 2021b). Monoclonal antibodies derived from COVID-19 convalescent patients are relatively safe and effective therapies with few side effects and quick preparation. Tai et al. exhibited that SARS-CoV RBD-based mouse monoclonal antibodies (mAbs), such as 7B11, can prevent SARS-CoV-2 from binding to ACE2 (Tai et al., 2020; Min and Sun, 2021). Cao et al. isolated highly effective monoclonal neutralizing antibodies from antigen-enriched B cells in convalescent-stage plasma and investigated their high affinity for RBD and potent neutralizing effect against the virus (Cao et al., 2020). Rogers et al. demonstrated that monoclonal antibodies were effective in neutralizing SARS-CoV-2 in Syrian hamsters (Rogers et al., 2020). Van et al. found that monoclonal antibodies C135-LS and C-144-LS can ameliorate clinical symptoms, reduce respiratory viral replication, and inhibit the production of pulmonary inflammation in Rhesus monkeys infected with SARS CoV-2 (Bueno et al., 2021; Van Rompay et al., 2021). Kim et al. tested the therapeutic effect of CT-P59 in three animal models (Ferret, Hamster, and Rhesus Monkey), and discovered that it significantly decreased SARS CoV-2 virus titer and alleviated clinical symptoms (Kim et al., 2021). Simone et al. demonstrated that monoclonal antibody MAD0004J08 induces SARS-CoV-2 neutralizing antibody titers in healthy adults on a single intramuscular injection that exceed those elicited by infection and vaccination (Bueno et al., 2021). Most importantly, Min et al. also comprehensively summarized REGN10987, REGN10933 (NCT04519437), VIR-7831 (GSK 4182136), AZD7442 (COV2-2196 and COV2-2130), LY-CoV555, among which exhibits neutralizing activity against SARS-CoV-2 and improves the recovery procession of patients with COVID-19 (Min and Sun, 2021). These studies will contribute to the advancement of monoclonal neutralizing antibodies into the clinic. Many monoclonal antibodies against SARS-CoV-2 are currently in clinical trials. LY-COV555, REGN-COV2 (composed of REGN10987 and REGN10933 monoclonal antibodies), and CT-P59 have been granted Emergency Use Authorization (EUA). TY027, BRII-196, BRII-198, and SCTA01 are in phase III clinical trials (Baral et al., 2021).

Additionally, monoclonal antibodies against SARS-CoV-2 can be generated using the hybridoma approach (Chapman et al., 2021; Tabll et al., 2021). However, due to the numerous adverse reactions caused by monoclonal antibodies and the difficulty of the technique, the hybridoma antibodies have not been used to treat patients with COVID-19. The hybridoma technique is the traditional method for isolating monoclonal antibodies. Because the natural process of somatic hypermutation and affinity maturation occurring within the host, the full-length bivalent antibodies extracted using this approach exhibit excellent affinity and neutralizing capacity. However, protein drugs may be immunogenic and may result in the formation of the anti-drug antibodies (ADAs) in humans. ADAs can impair the effectiveness of medications, cause allergic reactions, and even put lives at risk. Furthermore, the primary prerequisite for hybridoma technique is that the host animal be immunized with the disease in order to induce specific monoclonal antibodies, which is not ethical in humans. Due to the lengthy process of screening, gene cloning, and humanization of antibodies following recombinant antibody preparation, the hybridoma pathway is mainly employed for diagnostic antibodies and has not been used for COVID-19 therapeutic antibodies.

To create specific monoclonal antibodies, the spleen cells of immunized mice are fused with myeloma cells. However, due to the rejection response induced by the animal antibodies, the resulting monoclonal antibodies are not acceptable for direct use in humans. Antibodies must therefore be humanized. Humanized antibodies are mouse monoclonal antibodies that have been partially (CH and CL area) or completely encoded by human antibody genes *via* gene cloning or DNA recombination technology. The majority of its amino acid sequence is replaced with human sequences, keeping the affinity and specificity of the parent mouse monoclonal antibody, but decreasing its heterogenicity, which is advantageous for human application. Chimeric antibodies, modified antibodies, and fully humanized antibodies are all examples of humanized antibodies.

Additionally, there are many important human monoclonal antibodies that were granted EUA in the US. REGN-COV2, an antibody cocktail containing two noncompeting, neutralizing human IgG1 antibodies that target the receptor-binding domain of the SARS-CoV-2 spike pro tein, thereby preventing viral entry into human cells through the angiotensin-converting enzyme 2 (ACE2) receptor (Baum et al., 2020; Hansen et al., 2020). In a phase 1–3 trial involving nonhospitalized patients with the original strain of COVID-19, it showed reduced viral load, with a greater effect in patients whose immune response had not yet been initiated or who had a high viral load at baseline with good safety (Weinreich et al., 2021). On Oct. 14, 2021, the REGEN-COV monoclonal antibody therapy was approved by FDA and EMA (European Medicines Agency) for post-exposure prophylaxis (prevention). After the approvement, Shigehisa Arikawa etc. reported that REGN-COV2 was effective for the hemodialysis patients in a case series of 20 patients (Copin et al., 2021).

Bamlanivimab/etesevimab was another neutralizing monoclonal antibody which isolated from convalescent plasma obtained from patients with Covid-19 in the United States and China, which could lead to a lower incidence of Covid-19–related hospitalization and death than did placebo and accelerated the decline in the SARS-CoV-2 viral load among high-risk ambulatory patients (Dougan et al., 2021). An observational prospective study showed that in patients infected by the SARS- CoV-2 Gamma variant, Bamlanivimab/etesevimab should be used with caution because of the high risk of disease progression (Falcone et al., 2021). However, because data show these treatments are highly unlikely to be active against the omicron variant, which is circulating at a very high frequency throughout the world (Epstein, 2022).

In fact, new drugs are always a matter of life and death. Sotrovimab is a newly developed monoclonal antibody-based medicine which stops the action of the virus that causes COVID-19. In a multinational, double-blind, randomized, placebo-controlled clinical trial, unfortunately neither sotrovimab nor BRII-196 plus BRII-198 showed efficacy for improving clinical outcomes among adults hospitalized with COVID-19 (Group AC-TfIwC-S, 2022).

Tixagevimab and Cilgavimab (Evusheld) were long-acting monoclonal antibodies to be administered concomitantly by IM injection for pre-exposure prophylaxis of COVID-19 in persons ≥12 years old who weigh ≥40 kg and have either a history of severe allergy that prevents their vaccination against COVID-19 or moderate or severe immune compromise (Abramowicz et al., 2022). They are the first drugs to be authorized by the FDA for pre-exposure prophylaxis of COVID-19. The U.S. Food and Drug Administration (FDA) has revised its emergency use authorization (EUA) for AstraZeneca’s Evusheld to a higher dose to be effective in the prevention of COVID-19. Evusheld earned the FDA’s approval in December 2021 following positive results from its PROVENT trial, which hit an 83% efficacy compared to placebo during a six-month study. AstraZeneca also conducted a Phase III TACKLE trial for outpatient participants, which delivered positive high-level results (Abramowicz et al., 2022). These drugs escape the neutralization activity against the Omicron SARS-CoV-2 variant (B.1.1.529), according to virus neutralization data from two vitro studies (VanBlargan et al., 2022).

### 3.4. Preparation of recombinant drugs

Historically, recombinant drugs have been produced primarily through cell hybridization (mAb), or molecular cloning, and genetic engineering (recombinant antibody). The development of mAbs and their remarkable properties (high specificity, natural protein products, and others), have resulted in their extensive usage in disease research, diagnosis, and treatment. Recombinant antibodies can be produced in as little as a few weeks, and their economics and efficiency make them ideal for high-throughput production. The platform for protein expression is stable and repetitive, with a large selection space, and it is capable of expressing proteins in fragments or multiple hosts. Additionally, it may be able to address the immunogenicity of monoclonal antibodies obtained from animals and animal ethics.

There are two main methods for producing recombinant antibodies. To obtain protein products, monoclonal B cells are extracted, sequenced, and screened for target antibodies. After creating specific antibodies by recombination, a humanization step is required to obtain the therapeutic antibodies. On the other hand, polyvalent nanobodies are obtained *via* genetic engineering and phage display screening from camels or other transgenic animals.

Moreover, transgenic hACE2 mice nowadays have been widely accepted as a reliable and valuable model for studying the pathology and pathogenesis of SARS-CoV-2 infection. These genetically modified mice express human ACE2, the receptor utilized by the virus to enter host cells, and thus, they are able to more accurately mimic the lower respiratory tract infection seen in humans (Veenhuis and Zeiss, 2021; Yang et al., 2022). On the other hand, wild-type mouse ACE2 has been found to be insufficient in replicating the same degree of respiratory tract infection as seen in human beings, thus highlighting the importance of using transgenic hACE2 mice as a model for studying the virus (Yang et al., 2007). In addition to mice, hamsters have also been identified as a potential animal model for SARS-CoV-2 infection (Pandey et al., 2021). Studies have shown that hamsters can be infected with the virus and develop symptoms similar to those seen in humans, including lung pathology and viral replication in the respiratory tract (Becker et al., 2021; Bednash et al., 2022).

#### 3.4.1. Monoclonal B cell isolation and sequencing

After immunizing animals, plasmacytes will be isolated and sorted using flow cytometry. Cells isolated are screened for the target, amplified, and cloned into the expression vector. The gene with highest level of expression is identified and sequenced using enzyme-linked immunosorbent assay (ELISA). Finally, utilizing *in vitro* recombinant technology, recombinant antibodies will be created.

#### 3.4.2. Nanobodies

Nanobodies are a type of antibody found exclusively in camelids and cartilaginous fish such as sharks (Voss, 2021), which has a single variable heavy chain region (VHH). It is naturally deficient in the light chain when compared to other antibodies (Jovcevska and Muyldermans, 2020). Due to the lack of a VL structure, they are hydrophilic. Nanobodies are tiny and stable at high temperatures or extreme pH. VHH preserves intact antigenic specificity at around 15 kDa, despite its molecular weight being only 1/10 that of conventional antibodies (Xue et al., 2016). This property enables nanobodies to bind to epitopes not normally accessible by conventional antibodies, such as conserved domains that are typically masked by glycan (Xu et al., 2021). Moreover, nanobodies are perfectly compatible with phage display.

Nanobodies do not contain the Fc segment seen in traditional antibodies. Hence there is no reason to be concerned about the complement reaction caused by the Fc segment, which results in some unusual features. Nanobodies can be used to construct multiple molecular structures that neutralize cytokines and soluble proteins. On the one hand, as an intracellular antibody, it is capable of recognizing and neutralizing the virus’s unique structure, so aiding the host’s immune defense. On the other hand, it can be built into bifunctional or bispecific antibodies for disease targeting. Nanobodies can be genetically modified to form polymers, significantly increasing the antibody’s ability to bind antigen (Atagenix, 2021).

Xu et al. inserted the camel VHH gene into the mouse genome. Three tandem nanobodies were generated and fused with the human antibody Fc domain using phage display technique and genetic engineering modification. The tests demonstrate that the polyvalent nanobody (recognizing different sites) not only neutralizes SARS-CoV-2 but also prevents it from escaping (Xu et al., 2021). The Fc domain is responsible for the antibody’s distribution throughout the body. It not only prolongs the antibody’s lifespan but also promotes its interaction with other immune components. As a result, using the Fc domain-bound form should aid in antigen binding (Voss, 2021).

Due to the nanobody’s size and solubility, it can be administered directly by inhalation to target key initial sites of viral replication in the respiratory. When concerning the size implications, the key advantages of nanobodies is their small size, which allows them to access anatomical regions that are otherwise inaccessible to larger, heterotetrameric antibodies (Muyldermans, 2013). This is particularly relevant when it comes to the brain, which is protected by the blood–brain barrier (BBB). The BBB is a highly selective barrier that prevents the majority of molecules, including antibodies, from crossing into the brain. However, because nanobodies are small enough to traverse the BBB, they have the potential to be used for the diagnosis and treatment of brain-related diseases (Jovcevska and Muyldermans, 2020). Furthermore, phage display technology can be used to screen nanobodies with non-overlapping or slightly overlapping action sites that can be used as adjuncts to monoclonal antibodies targeting RBD.

#### 3.4.3. Phage display antibody library

Phage display technology offers the construction and manipulation of antibody library genes in ways that other antibody isolation technologies do not. This is a powerful tool for creating effective human antibodies. The procedure involves isolating antibodies from B cells and using PCR to amplify their heavy chain (VH) and light chain (VL) genes. After that, the phage carrier is constructed, replicated, translated, and assembled into infectious phages. Then, the serum would be analyzed to determine if it hand the necessary antibodies (Ali et al., 2020). The Escherichia coli expression system used for phage display makes this technique more cost-effective and scalable than other eukaryotic display methods.

The modified phage has the antibody gene and antibody molecules are expressed on its surface. The antigen–antibody specific combination can be used to rapidly screened for the target antibody, which can subsequently be cloned and amplified. If the antibody gene is expressed as secreted, soluble fragments of the antibody can be obtained. By combining VH and VL randomly, a combinatorial antibody library can be constructed. Perween et al. introduced the OCHRE (TAA) codon at the junction of single-chain variable fragment (scFv) and PIII gene, thereby speeding up the initial screening of scFv. They identified a scFv -- B8 that binds exclusively and with nanomolar affinity to SARS-CoV-2 RBD (Perween et al., 2021).

## 4. Functional evaluation

Antibody affinity, blocking activity, and virus-neutralizing activity are all evaluated *In vitro* for nAb. Various approaches, such as ELISA, surface plasm resonance, and high-throughput analysis using flow cytometry and recombinant cells expressing SARS-CoV-2 antigen, can be used to assess nAb affinity and blocking activity (Rogers et al., 2020; Shi J. et al., 2020). The infection inhibition test can be used to measure the neutralizing activity of antibodies. At present, the most frequently used procedures are PRNT with live virus and microcell neutralization tests using cytopathic effects detection. Cytopathic effects in cells can be noticed with the naked eyes or in the form of virus spots that can be recognized by immunofluorescence staining after SARS-CoV-2 infection (Ju et al., 2020). Another *in vitro* assay system based on a pseudovirus can be utilized to determine coronavirus antibody levels. For example, Chinese researchers compared and evaluated the neutralizing activity of different nAbs against SARS-CoV-2 using a VSV pseudovirus expressing S protein and the infected cell line Huh-7 (Shi J. et al., 2020). The pseudovirus incorporates SARS-CoV-2 spike into the lentiviral/retroviral backbones and contains reporter genes such as GFP or luciferase that can be used to indicate infection (Ju et al., 2020; Xiong et al., 2020). By contrast, such technology provides a number of advantages, including a high throughput and the absence of the requirement for experimental facilities. On pseudoviruses, the shape and quantity of S proteins may differ from those on the real virus. Generally speaking, the pseudovirus test is utilized initially to screen for lead antibodies at a high throughput, and then the live virus test is used to further verify the lead candidates.

In terms of *in vivo* evaluation, commonly used animal models to simulate SARS-CoV-2 infection are mice/rats, ferrets, and non-human primates. Sia et al. (2020) reported that SARS-CoV-2 can be effectively transmitted from infected hamsters to uninfected hamsters through direct contact and aerosol. Neutralization antibodies were detected in all recovered animals. Chandrashekar et al. (2020) demonstrated that infected rhesus monkeys did not revert to infection after a short period of time. Shi R. et al. (2020) found that the antibody CB6-LALA was effective at preventing and treating SARS-CoV-2 in rhesus monkeys, including virus titer reduction and alleviation of related lung pathology. Clinical research for this antibody CB6-LALA treatment is presently underway.

Further, the efficacy of antibodies is a crucial aspect in determining the effectiveness of a therapeutic treatment. In particular, the ability of these molecules to bind to and neutralize their intended target antigen is of paramount importance. However, it is not only the efficacy of the antibodies that is important, but also the patient’s tolerance to the treatment. Patient tolerance, in this context, refers to the ability of a patient to endure the treatment without experiencing severe side effects. While a treatment may have a high efficacy in binding to and neutralizing its intended target antigen, it may not be suitable for a given patient if they are unable to tolerate it. In the case of the study by Focosi, the use of cilgavimab plus tixagevimab monoclonal antibody cocktail may have a high efficacy in binding to and neutralizing its intended target antigen (Focosi and Casadevall, 2022). However, it may not be a suitable treatment option for certain patients as its utilization may lead to an immune response against the therapeutic, resulting in the formation of immune complexes that may exacerbate the disease. This highlights the importance of considering both efficacy and patient tolerance in the selection of an appropriate treatment.

## 5. Future challenges

Despite its established safety and efficacy, neutralizing antibody therapy continues to confront numerous obstacles. Human immunity to SARS-CoV-2 is a complicated process that cannot be effectively treated with neutralizing antibodies alone. It must be viewed in its entirety. SARS-CoV-2 immune escape, cytokine storm, the ADE, and other factors can all reduce the efficacy of neutralizing antibody therapy. Furthermore, some immune drugs, such as convalescence plasma, IVIG, and monoclonal antibodies, are not as widely used or as effective in clinical treatment as expected.

The long-term effects of therapeutics for SARS-CoV-2 are not yet fully understood, as the virus variants and the therapeutics are both relatively new and continually updated. However, some studies suggest that individuals who recover from SARS-CoV-2 infection may have some level of immunity to the virus, although it is not yet clear how long this immunity lasts (Pilz et al., 2022; Willyard, 2022; Zheng et al., 2022). It has been observed that some people who have recovered from COVID-19 may still be at risk of subsequent infections with the same or different strains of SARS-CoV-2 (Flacco et al., 2022; Liu et al., 2022). Liang et al. (2022) and Guo et al. (2022) have shown that some patients who have recovered from COVID-19 may have a reduced level of antibodies against the virus after several months. Additionally, some studies have suggested that the presence of antibodies does not guarantee protection against reinfection, as there have been reports of people testing positive for the virus again after recovering from an initial infection (Negi et al., 2022; Nguyen et al., 2022; Ruiz-Galiana et al., 2022). Overall, more research is needed to fully understand the long-term effects of therapeutics for SARS-CoV-2 and the level of immunity they provide. It is possible that individuals who recover from SARS-CoV-2 infection may be at risk of subsequent infections with the same or different strains of the virus, and that the immunity provided by therapeutics may not be long-lasting.

In terms of immunological drugs, the following sections will discuss the difficulties and prospects for neutralizing antibody therapy.

### 5.1. Challenges of convalescent plasma

Many scholars cast doubt on the safety and efficacy of CP, raising concerns about the suitability of plasma donors, differences in the levels of nAbs in convalescents, antibody immune maintenance levels, immune deficits, the effect of component and blood coagulation factor, and new virus variants. These factors influence the therapeutic effect of CP to some extent. Studies have noted that CP with a high titer of nAbs does not benefit marked inpatients 10 days after symptom onset, presumably because the inpatients already have a high titer. As a result, it is recommended that CP be used earlier in the course of disease (Gharbharan et al., 2021). Even if there is a significant nAbs titer in CP, it has no effect on the antiviral activity, i.e., virus clearance rate, of life-threatening SARS-CoV-2 patients. It is challenging to deliver CP to critically ill patients in the advanced stages of the disease in order to reverse the hyperinflammatory state and improve clinical status (Abuzakouk et al., 2021). Terpos et al. demonstrated that, while nAbs directed against S protein were more persistent than those directed against N protein, nAbs in CP lacked long-term persistence, requiring long-term donor follow-up (Terpos et al., 2021).

As a result, some studies have proposed solutions for CP and conducted exploratory experiments. Many researchers have advocated developing adequate detection methods to evaluate the quality of CP, and treating it technically to enhance its therapeutic impact (von Rhein et al., 2021). If CP is to be used effectively in clinical practice, its capacity for neutralization must be accurately evaluated. Bernd et al. evaluated the neutralization potential of 111 convalescent plasma donors 7 months after diagnosis using three neutralization platforms (wild-type virus, pseudovirus, and surrogate neutralization test platform). Within a range of options, the detection performance parameters of these three test systems were satisfactory. Patients benefit considerably from CP with high titers of nAbs, and these findings may aid in the development of a test strategy for rapid selection of high-titer convalescent plasma products (Jahrsdorfer et al., 2021). A number of CP technologies are being developed that, when utilized properly, might offer patients new hope. Riboflavin and ultraviolet pathogen reduction (R + UV PRT) have been shown in studies to reduce the risk of pathogen transmission during blood transfusions while also having therapeutic effects on blood coagulation factors. More critically, R + UV PRT had a little effect on immunoglobulin concentration (including the IgG subclass), preserving the neutralizing ability of CP (Yonemura et al., 2021). By undergoing immunoadsorption, several researchers have developed that collect concentrated immunoglobulins rather than whole plasma units. The immunoglobulin concentration is 10 times that of peripheral blood and may produce more than eight times the number of neutralizing antibodies produced by a single plasma unit, implying that this pattern contributes to higher nAb levels and effectiveness of CP (Rothenburg et al., 2021).

### 5.2. Challenges of intravenous immunoglobulin

Although IVIG has been used to treat severe viral, bacterial, or immunodeficiency diseases, its efficacy in clinical trials for SARS-CoV-2 is unknown, and there are few randomized controlled trials and observational studies with control groups. IVIG can result in ADE, transfusion-associated acute lung injury (TRALI), as well as thrombus formation. IVIG formulations are regularly marketed, and noted shortcomings include high cost, limited potency, impurity problems, insufficient supply batches, and batch variability. Furthermore, IVIG is mainly prepared through industrial manipulation, virus inactivation, and physical or chemical virus and bacterium eradication. As a result, different drug properties such as osmotic pressure and IgA content are altered, affecting both safety and efficacy. IVIG treatment still necessitates close monitoring (Perricone et al., 2021).

In addition to standard care, IVIG therapy did not significantly improve COVID-19 in patients who were not critically ill, but this finding has not been confirmed in controlled trials yet (Huang et al., 2021). Independent factors such as male gender, elderly age (60 years), hypertension, severe illness, and IVIG significantly prolong the duration of SARS-CoV-2 virus clearance (15 days), showing that IVIG may increase the risk of viral clearance, reduce treatment effectiveness, and prolong the course of disease in patients (Chen X. et al., 2021). Hou et al. demonstrated that adjuvant IVIG therapy had no effect on in-hospital mortality or the need for mechanical ventilation in patients with severe COVID-19, implying that immunoglobulin may not be useful in these patients (Hou et al., 2021).

According to research, anti-SARS-CoV-2 IgG detection in the serum of IVIG treatment patients is useful for assessing the nAbs level and therapeutic efficacy of IVIG preparations (Dalakas et al., 2021). A recent study developed hyperimmune intravenous immunoglobulin (C-IVIG), which is considerably superior for patients with no immediate and serious adverse drug reactions. C-IVIG may help suppress lung injury by the virus, complement activation, and cytokine storm, among other effects, hence improving survival rates in severely and critically ill patients (Ali et al., 2021).

### 5.3. Challenges of monoclonal antibodies

#### 5.3.1. Virus mutation

Monoclonal antibodies are directed against a specific site on the virus, whereas SARS-CoV-2 is constantly mutating. The Alpha variant (UK variant), the Beta variant (SA variant), the Gamma variant found in Brazil, and particularly the Delta variant found in India. The variant has a significant impact on the efficacy of mAb and even escape from them (Corti et al., 2021). To address this issue, researchers are working on developing mAb directed against the conserved domain of SARS-CoV-2 and combining multiple mAbs that target different sites into a cocktail of antibodies against viral mutations.

#### 5.3.2. Adverse drug reaction

In COVID-19 patients, mAbs have the potential to cause a wide range of serious adverse drug reactions. In patients who died from Sars-CoV, Liu et al. discovered a violent neutralizing response and accumulation of pulmonary inflammation, implying that nAb may cause fatal acute lung injury (Liu et al., 2019). It is postulated that mAb may contribute to adverse reactions such as lung injury in COVID-19 patients. Furthermore, mAbs may be immunogenic, leading in the production of anti-drug antibodies (ADAs), which reduce mAbs efficacy and elicit undesirable immunological reactions. Vaisman et al. discovered that monoclonal antibodies can be immunogenic regardless of their source (Vaisman-Mentesh et al., 2020). Additional study on the immunogenicity of mAbs is required to improve their safety and efficacy in people.

#### 5.3.3. Antibody dependence effect

Monoclonal antibodies may also raise the likelihood of antibody dependency effect (ADE). ADE facilitates viral infection and impairs the immune system’s function. Due to frequent mutation and high infectivity of SARS-CoV-2, and the multiple reactions generated by mAbs *in vivo* and the fact that SARS-CoV-2 has not been confirmed to cauce ADE *in vivo*, it is unlikely that it will in the future. Wan et al. discovered that SARS-CoV and MERS-CoV cause ADE both *in vivo* and *in vitro* and that the mechanism of ADE was mediated by antibody Fc (Vaisman-Mentesh et al., 2020). Wang et al. confirmed that the MW05 and MW07 (both are mAbs) induce ADE effects *in vitro via* the Fc segment (Wang et al., 2020). These findings suggest that mAbs may cause ADE, which is most likely induced by Fc. To avoid the occurrence of ADE, mAb preparation should consider coating or modifying the Fc segment without affecting the antibody’s activity.

#### 5.3.4. Individual differences and costs

Monoclonal antibodies exert a variety of effects depending on the individuals and stages of viremia. Pharmacokinetics discrepancy complicates determining mAb dosage (Johnson et al., 2021), whether monoclonal antibodies are suitable for all COVID-19 patients, and achieving precise treatment and customized medication. Furthermore, mAb preparation technology is complicated, requiring a great deal of time, labor, and money. Because the annual cost of treating cancer patients with mAb is around $35,000 per patient, it is challenging to reach low-income populations with these pricey medicines. Without external financial support, many low-income countries would struggle to handle the strain.

#### 5.3.5. Impact on antibody treatment

The SARS-CoV-2 virus is composed of four structural proteins, of which the spike glycoprotein is critical for viral attachment, fusion, and entry and therefore is a key target for anti-bodies and vaccines. The RBD unit of the spike protein mediates viral entry by binding to the angiotensin-converting enzyme 2 on the host cell, which is a cell receptor expressed by lung, gastrointestinal tract, nasal mucosa cells, and in particular neurons, astrocytes and oligodendrocytes with intricate interactions and delicate balance making the brain an attractive target for viral infections. Recent research has highlighted the vulnerability of these cells to viral infections, particularly those caused by coronaviruses. Studies have shown that the spike protein of the virus binds to the ACE2 receptor, which is present on the surface of neurons, astrocytes, and oligodendrocytes (Conte, 2021; Mahalakshmi et al., 2021; Frank et al., 2022). This binding leads to the internalization of the virus, leading to its replication and spread within the brain and their damage can lead to a host of neurological and cognitive disorders, such as encephalitis, dementia, and neurodegeneration (Verkhratsky et al., 2020).

Naturally occurring variants in viruses are common with some mutations altering binding affinity and infectivity. The recently emerged Omicron (B.1.1.529) variant, a highly mutated SARS-CoV-2 variant, classified as VOC by World Health Organization (WHO) on November 26, 2021, has now spread to nearly 150 countries and territories, owing to its very high transmissibility and infectivity (Mohapatra et al., 2022). This variant exhibited more than 30 amino acid mutations in the spike protein. This mutation rate is exceeding the other variants by approximately 5–11 times in the receptor-binding motif of the spike protein. Omicron (B.1.1.529) variant might have enhanced transmissibility and immune evasion. This new variant can reinfect individuals previously infected with other SARS-CoV-2 variants. Some of the crucial mutations that are detected in the receptor-binding domain of the Omicron variant have been shared by previously evolved SARS-CoV-2 variants. Based on the Omicron mutation profile in the receptor-binding domain and motif, it might have collectively enhanced or intermediary infectivity relative to its previous variants. Due to extensive mutations in the spike protein, the Omicron variant might evade the immunity in the vaccinated individuals (Kannan et al., 2021). Omicron has shown immune escape from neutralizing antibodies generated through previous infection or vaccination.It could evade the protection provided by mAbs being used in clinics for treating coronavirus disease 2019 (COVID-19) patients.

Particularly, variants such as Omicronhave has high transmissibility and infectivity, and Omicron possessing higher immune evading properties, results in a reduction in vaccine-induced immunity with lowering efficacy of the available vaccines. Omicron can also overpower the protection rendered by antibodies-based immunotherapies through escaping the neutralization potential of therapeutic monoclonal antibodies (mABs), therefore some mAbs currently available for use in clinics may lose efficacy and will not be useful in treating Omicron-infected patients.

VanBlargan et al. have tested the anti-RBD mAbs which are in clinical use by AstraZeneca, Vir Biotechnology, Eli Lilly, Regeneron, and Celltrion for their ability to neutralize an infectious Omicron (B.1.1.529) isolate and in this per report, most of the monoclonal antibodies demonstrated a complete absence of neutralizing activity against Omicron in both the Vero-hACE2-TMPRSS2 and Vero-TMPRSS2 cell lines.

In such an adverse situation, newer strategies to develop next-generation mutation-proof SARS-CoV-2 vaccines and designing more effective and additional mAbs are required that would be more robust in countering highly mutated variants. Additionally, exploring more effective drugs and treatable options are the need of the hour (Mohapatra et al., 2022).

In spite of the anti-RBD mAbs tested were able to neutralize the Omicron strain to varying degrees, with some showing higher efficacy than others, omicron is able to evade most therapeutic monoclonal antibodies, and largely evade vaccine-elicited antibodies. Current treatments for omicron primarily use booster doses to increase the level of antibody protection in COVID-19 vaccinated individuals. In the future, it is hoped that through the research of scientists, better specific targeted drugs for the treatment of SARS-COV-2 will be found.

## 6. Conclusion

The COVID-19 pandemic has taken the world by storm causing widespread suffering and economic disruption. Despite this, the global effort to combat SARS-CoV-2 has seen remarkable progress in recent years. One of the most impressive affairs of the pandemic highlights the impressive advancements in clinical therapy, which has been the development of highly effective vaccines that have been instrumental in mitigating the severity of the disease and saving countless lives. Another important advance has been the development of antiviral drugs, such as remdesivir, which has been shown to reduce the length of hospital stays for patients with COVID-19. In addition, the use of monoclonal antibodies has been shown to be effective in preventing hospitalization for high-risk patients. However, several challenges in the fight against SARS-CoV-2 have also been faced by the researchers. The biggest one is the lack of comprehensive, accurate, up-to-date data on the SARS-CoV-2 shared by the global. This has made it difficult for researchers to fully rapidly understand the mechanisms of SARS-CoV-2 and develop effective treatments and preventions. In addition, such virus’s ability to swiftly mutate has presented challenges for developing long-term solutions. Nevertheless, with continued support and efforts in this realm, we can overcome these challenges and find effective solutions to the COVID-19 pandemic.

## Author contributions

ML, HG, ZL, and LL wrote the main manuscript text. QL, YM, HgC, and BL collected and reviewed many articles from literature. SY and HiC did some updating of the latest research studies. PZ and BS conceptualize and supervised the work. All authors contributed to the article and approved the submitted version.

## Funding

Study supported by Zhongnanshan Medical Foundation of Guangdong Province (nos. ZNSA-2021005 and ZNSA-2020001), State Key Laboratory of Respiratory Disease, Guangdong-Hong Kong-Macao Joint Laboratory of Respiratory Infectious Disease (no. GHMJLRID-Z-202102), Emergency key project of Guangzhou Laboratory (no. EKPG21-30-2), and Cultivation Project of the First Affiliated Hospital of Guangzhou Medical University (no. ZH202105).

## Conflict of interest

The authors declare that the research was conducted in the absence of any commercial or financial relationships that could be construed as a potential conflict of interest.

## Publisher’s note

All claims expressed in this article are solely those of the authors and do not necessarily represent those of their affiliated organizations, or those of the publisher, the editors and the reviewers. Any product that may be evaluated in this article, or claim that may be made by its manufacturer, is not guaranteed or endorsed by the publisher.

## References

[ref13] Abramowicz M. Zucotti G. Pflomm M. (2022). Tixagevimab and Cilgavimab (Evusheld) for pre-exposure prophylaxis of COVID-19. JAMA 327, 384–385. doi: 10.1001/jama.2021.24931, PMID: 35076671

[ref1] Abuzakouk M. Saleh K. Algora M. Nusair A. Alameri J. Alshehhi F.. (2021). Convalescent plasma efficacy in life-threatening COVID-19 patients admitted to the ICU: a retrospective cohort study. J. Clin. Med. 10. doi: 10.3390/jcm10102113, PMID: 34068847 PMC8153619

[ref2] Adams S. H. Park M. J. Schaub J. P. Brindis C. D. Irwin C. E. Jr. (2020). Medical vulnerability of young adults to severe COVID-19 illness—data from the National Health Interview Survey. J. Adolesc. Health 67, 362–368. doi: 10.1016/j.jadohealth.2020.06.025, PMID: 32674964 PMC7355323

[ref3] Afar D. E. Vivanco I. Hubert R. S. Kuo J. Chen E. Saffran D. C.. (2001). Catalytic cleavage of the androgen-regulated TMPRSS2 protease results in its secretion by prostate and prostate cancer epithelia. Cancer Res. 61, 1686–1692. 11245484

[ref4] Alfaleh M. A. Zawawi A. Al-Amri S. S. Hashem A. M. (2022). David versus goliath: ACE2-fc receptor traps as potential SARS-CoV-2 inhibitors. MAbs Taylor & Francis. 10.1080/19420862.2022.2057832 PMC8986284 35380919

[ref5] Ali S. Uddin S. M. Shalim E. Sayeed M. A. Anjum F. Saleem F.. (2021). Hyperimmune anti-COVID-19 IVIG (C-IVIG) treatment in severe and critical COVID-19 patients: a phase I/II randomized control trial. EClinicalMedicine 36:100926. doi: 10.1016/j.eclinm.2021.100926, PMID: 34109306 PMC8177439

[ref6] Ali M. G. Zhang Z. Gao Q. Pan M. Rowan E. G. Zhang J. (2020). Recent advances in therapeutic applications of neutralizing antibodies for virus infections: an overview. Immunol. Res. 68, 325–339. doi: 10.1007/s12026-020-09159-z, PMID: 33161557 PMC7648849

[ref7] Andabaka T. Nickerson J. W. Rojas-Reyes M. X. Rueda J. D. Bacic Vrca V. Barsic B. (2013). Monoclonal antibody for reducing the risk of respiratory syncytial virus infection in children. Cochrane Database Syst. Rev. CD006602. doi: 10.1002/14651858.CD006602.pub4, PMID: 23633336

[ref8] Anderson E. M. Goodwin E. C. Verma A. Arevalo C. P. Bolton M. J. Weirick M. E.. (2021). Seasonal human coronavirus antibodies are boosted upon SARS-CoV-2 infection but not associated with protection. Cells 184, 1858–64 e10. doi: 10.1016/j.cell.2021.02.010, PMID: 33631096 PMC7871851

[ref9] Andreano E. Nicastri E. Paciello I. Pileri P. Manganaro N. Piccini G.. (2021). Extremely potent human monoclonal antibodies from COVID-19 convalescent patients. Cells 184, 1821–35 e16. doi: 10.1016/j.cell.2021.02.035, PMID: 33667349 PMC7901298

[ref10] Astronomo R. D. Lemos M. P. Narpala S. R. Czartoski J. Fleming L. B. Seaton K. E.. (2021). Rectal tissue and vaginal tissue from intravenous VRC01 recipients show protection against ex vivo HIV-1 challenge. J. Clin. Invest. 131. doi: 10.1172/JCI146975, PMID: 34166231 PMC8363291

[ref11] Atagenix (2021). Antibody discovery: Nanobodies.

[ref12] Augustin M. Schommers P. Stecher M. Dewald F. Gieselmann L. Gruell H.. (2021). Post-COVID syndrome in non-hospitalised patients with COVID-19: a longitudinal prospective cohort study. Lancet Regional Health-Europe 6:100122. doi: 10.1016/j.lanepe.2021.100122, PMID: 34027514 PMC8129613

[ref14] Azkur A. K. Akdis M. Azkur D. Sokolowska M. van de Veen W. Brüggen M. C.. (2020). Immune response to SARS-CoV-2 and mechanisms of immunopathological changes in COVID-19. Allergy 75, 1564–1581. doi: 10.1111/all.14364, PMID: 32396996 PMC7272948

[ref15] Baral P. K. Yin J. James M. N. G. (2021). Treatment and prevention strategies for the COVID 19 pandemic: a review of immunotherapeutic approaches for neutralizing SARS-CoV-2. Int. J. Biol. Macromol. 186, 490–500. doi: 10.1016/j.ijbiomac.2021.07.013, PMID: 34237371 PMC8256663

[ref16] Bartleson J. M. Radenkovic D. Covarrubias A. J. Furman D. Winer D. A. Verdin E. (2021). SARS-CoV-2, COVID-19 and the aging immune system. Nat. Aging 1, 769–782. doi: 10.1038/s43587-021-00114-7, PMID: 34746804 PMC8570568

[ref17] Baum A. Fulton B. O. Wloga E. Copin R. Pascal K. E. Russo V.. (2020). Antibody cocktail to SARS-CoV-2 spike protein prevents rapid mutational escape seen with individual antibodies. Science 369, 1014–1018. doi: 10.1126/science.abd0831, PMID: 32540904 PMC7299283

[ref18] Becker K. Beythien G. de Buhr N. Stanelle-Bertram S. Tuku B. Kouassi N. M.. (2021). Vasculitis and neutrophil extracellular traps in lungs of golden Syrian hamsters with SARS-CoV-2. Front. Immunol. 12:640842. doi: 10.3389/fimmu.2021.640842, PMID: 33912167 PMC8072219

[ref19] Bednash J. S. Kagan V. E. Englert J. A. Farkas D. Tyurina Y. Y. Tyurin V. A.. (2022). Syrian hamsters as a model of lung injury with SARS-CoV-2 infection: pathologic, physiologic, and detailed molecular profiling. Transl. Res. 240, 1–16. doi: 10.1016/j.trsl.2021.10.007, PMID: 34740873 PMC8562047

[ref20] Brodin P. (2021). Immune determinants of COVID-19 disease presentation and severity. Nat. Med. 27, 28–33. doi: 10.1038/s41591-020-01202-8, PMID: 33442016

[ref21] Brouwer P. J. M. Caniels T. G. van der Straten K. Snitselaar J. L. Aldon Y. Bangaru S.. (2020). Potent neutralizing antibodies from COVID-19 patients define multiple targets of vulnerability. Science 369, 643–650. doi: 10.1126/science.abc5902, PMID: 32540902 PMC7299281

[ref22] Brynjolfsson S. F. Sigurgrimsdottir H. Einarsdottir E. D. Bjornsdottir G. A. Armannsdottir B. Baldvinsdottir G. E.. (2021). Detailed multiplex analysis of SARS-CoV-2 specific antibodies in COVID-19 disease. Front. Immunol. 12:695230. doi: 10.3389/fimmu.2021.695230, PMID: 34177962 PMC8222737

[ref23] Bueno S. M. Abarca K. González P. A. Gálvez N. M. Soto J. A. Duarte L. F.. (2021). A single intramuscular injection of monoclonal antibody MAD0004J08 induces in healthy adults SARS-CoV-2 neutralising antibody titres exceeding those induced by infection and vaccination. MedRxiv. doi: 10.1101/2021.03.31.21254494, PMID: 35441164 PMC9016657

[ref24] Cao W. Liu X. Hong K. Ma Z. Zhang Y. Lin L.. (2021). High-dose intravenous immunoglobulin in severe coronavirus disease 2019: a multicenter retrospective study in China. Front. Immunol. 12:627844. doi: 10.3389/fimmu.2021.724379, PMID: 33679771 PMC7933558

[ref25] Cao Y. Su B. Guo X. Sun W. Deng Y. Bao L.. (2020). Potent neutralizing antibodies against SARS-CoV-2 identified by high-throughput single-cell sequencing of convalescent Patients' B cells. Cells 182, 73–84 e16. doi: 10.1016/j.cell.2020.05.025, PMID: 32425270 PMC7231725

[ref26] Chandrashekar A. Liu J. Martinot A. J. McMahan K. Mercado N. B. Peter L.. (2020). SARS-CoV-2 infection protects against rechallenge in rhesus macaques. Science 369, 812–817. doi: 10.1126/science.abc4776, PMID: 32434946 PMC7243369

[ref27] Chapman A. P. Tang X. Lee J. R. Chida A. Mercer K. Wharton R. E.. (2021). Rapid development of neutralizing and diagnostic SARS-COV-2 mouse monoclonal antibodies. Sci. Rep. 11:9682. 33958613 10.1038/s41598-021-88809-0 PMC8102525

[ref28] Chen X. Hu Mt W. Yang M. Ling J. Zhang Y. Deng L.. (2021). Risk factors for the delayed viral clearance in COVID-19 patients. J. Clin. Hypertens. 23, 1483–1489. doi: 10.1111/jch.14308, PMID: 34171164 PMC8420571

[ref29] Chen Y. Lear T. B. Evankovich J. W. Larsen M. B. Lin B. Alfaras I.. (2021a). A high-throughput screen for TMPRSS2 expression identifies FDA-approved compounds that can limit SARS-CoV-2 entry. Nat. Commun. 12:3907. 34162861 10.1038/s41467-021-24156-y PMC8222394

[ref30] Chen Y. Zhu L. Huang W. Tong X. Wu H. Tao Y.. (2021b). Potent RBD-specific neutralizing rabbit monoclonal antibodies recognize emerging SARS-CoV-2 variants elicited by DNA prime-protein boost vaccination. Emerg. Microbes Infect. 10, 1390–1403. doi: 10.1080/22221751.2021.1942227, PMID: 34120577 PMC8274519

[ref31] Cheng Z. J. Huang H. Zheng P. Xue M. Ma J. Zhan Z.. (2022). Humoral immune response of Sinopharm/BBIBP COVID-19 vaccination before and after the booster immunization. Allergy. doi: 10.1111/all.15271, PMID: 35255171 PMC9111230

[ref32] Cheng Z. J. Shan J. (2020). 2019 novel coronavirus: where we are and what we know. Infection 48, 155–163. doi: 10.1007/s15010-020-01401-y, PMID: 32072569 PMC7095345

[ref33] Conte C. (2021). Possible link between SARS-CoV-2 infection and Parkinson’s disease: the role of toll-like receptor 4. Int. J. Mol. Sci. 22:7135. doi: 10.3390/ijms22137135, PMID: 34281186 PMC8269350

[ref34] Copin R. Baum A. Wloga E. Pascal K. E. Giordano S. Fulton B. O.. (2021). The monoclonal antibody combination REGEN-COV protects against SARS-CoV-2 mutational escape in preclinical and human studies. Cells 184, 3949–61 e11. doi: 10.1016/j.cell.2021.06.002, PMID: 34161776 PMC8179113

[ref35] Corti D. Purcell L. A. Snell G. Veesler D. (2021). Tackling COVID-19 with neutralizing monoclonal antibodies. Cells 184, 3086–3108. doi: 10.1016/j.cell.2021.05.005, PMID: 34087172 PMC8152891

[ref36] Dalakas M. C. Bitzogli K. Alexopoulos H. (2021). Anti-SARS-CoV-2 antibodies within IVIg preparations: cross-Reactivities with seasonal coronaviruses, natural autoimmunity, and therapeutic implications. Front. Immunol. 12:627285. doi: 10.3389/fimmu.2021.627285, PMID: 33679770 PMC7925824

[ref37] De Giorgi V. West K. A. Henning A. N. Chen L. N. Holbrook M. R. Gross R.. (2021). Naturally acquired SARS-CoV-2 immunity persists for up to 11 months following infection. J. Infect. Dis. 224, 1294–1304. doi: 10.1093/infdis/jiab295, PMID: 34089610 PMC8195007

[ref38] Deng Q. Rasool R. U. Russell R. M. Natesan R. Asangani I. A. (2021). Targeting androgen regulation of TMPRSS2 and ACE2 as a therapeutic strategy to combat COVID-19. iScience 24:102254. doi: 10.1016/j.isci.2021.102254, PMID: 33681723 PMC7919514

[ref39] Dhochak N. Singhal T. Kabra S. Lodha R. (2020). Pathophysiology of COVID-19: why children fare better than adults? Indian J. Pediatrics 87, 537–546. doi: 10.1007/s12098-020-03322-y, PMID: 32410003 PMC7221011

[ref40] Di Gaetano S. Capasso D. Delre P. Pirone L. Saviano M. Pedone E.. (2021). More is always better than one: the N-terminal domain of the spike protein as another emerging target for hampering the SARS-CoV-2 attachment to host cells. Int. J. Mol. Sci. 22. doi: 10.3390/ijms22126462, PMID: 34208755 PMC8235207

[ref41] Dispinseri S. Secchi M. Pirillo M. F. Tolazzi M. Borghi M. Brigatti C.. (2020). Neutralizing antibody responses to SARS-CoV-2 in a COVID-19 recovered patient cohort and their implications. MedRxiv. doi: 10.1101/2020.11.27.20239616, PMID: 34013296 PMC8132271

[ref42] Dispinseri S. Secchi M. Pirillo M. F. Tolazzi M. Borghi M. Brigatti C.. (2021). Neutralizing antibody responses to SARS-CoV-2 in symptomatic COVID-19 is persistent and critical for survival. Nat. Commun. 12:2670. 33976165 10.1038/s41467-021-22958-8 PMC8113594

[ref43] Dong J. Huang B. Jia Z. Wang B. Gallolu Kankanamalage S. Titong A.. (2020). Development of multi-specific humanized llama antibodies blocking SARS-CoV-2/ACE2 interaction with high affinity and avidity. Emerg. Microbes Infect. 9, 1034–1036. doi: 10.1080/22221751.2020.1768806, PMID: 32403995 PMC8284970

[ref44] Dougan M. Nirula A. Azizad M. Mocherla B. Gottlieb R. L. Chen P.. (2021). Bamlanivimab plus Etesevimab in mild or moderate Covid-19. N. Engl. J. Med. 385, 1382–1392. doi: 10.1056/NEJMoa2102685, PMID: 34260849 PMC8314785

[ref45] Epstein M. (2022). A podcast discussing aldosterone and mineralocorticoid receptor antagonists in 2021: a paradigm shift. Diabetes Ther. 13, 583–588. doi: 10.1007/s13300-022-01236-w, PMID: 35294746 PMC8991300

[ref46] Eslami N. Aghbash P. S. Shamekh A. Entezari-Maleki T. Nahand J. S. Sales A. J.. (2022). SARS-CoV-2: receptor and co-receptor tropism probability. Curr. Microbiol. 79, 1–13. 10.1007/s00284-022-02807-7 PMC8923825 35292865

[ref47] Esmaeilzadeh A. Elahi R. (2021). Immunobiology and immunotherapy of COVID-19: a clinically updated overview. J. Cell. Physiol. 236, 2519–2543. doi: 10.1002/jcp.30076, PMID: 33022076 PMC7675260

[ref48] Esmaeilzadeh A. Rostami S. Yeganeh P. M. Tahmasebi S. Ahmadi M. (2021). Recent advances in antibody-based immunotherapy strategies for COVID-19. J. Cell. Biochem. 122, 1389–1412. doi: 10.1002/jcb.30017, PMID: 34160093 PMC8427040

[ref49] Falcone M. Tiseo G. Valoriani B. Barbieri C. Occhineri S. Mazzetti P.. (2021). Efficacy of Bamlanivimab/Etesevimab and Casirivimab/Imdevimab in preventing progression to severe COVID-19 and role of variants of concern. Infect. Dis. Ther. 10, 2479–2488. doi: 10.1007/s40121-021-00525-4, PMID: 34435337 PMC8386337

[ref50] Farcet M. R. Karbiener M. Schwaiger J. Ilk R. Kreil T. R. (2021). Rapidly increasing SARS-CoV-2 neutralization by intravenous immunoglobulins produced from plasma collected during the 2020 pandemic. J. Infect. Dis. 10.1093/infdis/jiaa593 PMC7546122 32941626

[ref51] Flacco M. E. Acuti Martellucci C. Baccolini V. De Vito C. Renzi E. Villari P.. (2022). Risk of reinfection and disease after SARS-CoV-2 primary infection: meta-analysis. Eur. J. Clin. Investig. 52:e13845. doi: 10.1111/eci.13845, PMID: 35904405 PMC9353414

[ref52] Focosi D. Casadevall A. (2022). A critical analysis of the use of Cilgavimab plus Tixagevimab monoclonal antibody cocktail (Evusheld™) for COVID-19 prophylaxis and treatment. Viruses 14:1999. doi: 10.3390/v14091999, PMID: 36146805 PMC9505619

[ref53] Frank M. G. Nguyen K. H. Ball J. B. Hopkins S. Kelley T. Baratta M. V.. (2022). SARS-CoV-2 spike S1 subunit induces neuroinflammatory, microglial and behavioral sickness responses: evidence of PAMP-like properties. Brain Behav. Immun. 100, 267–277. doi: 10.1016/j.bbi.2021.12.007, PMID: 34915155 PMC8667429

[ref54] Gharbharan A. Jordans C. C. E. GeurtsvanKessel C. den Hollander J. G. Karim F. Mollema F. P. N.. (2021). Effects of potent neutralizing antibodies from convalescent plasma in patients hospitalized for severe SARS-CoV-2 infection. Nat. Commun. 12:3189. 34045486 10.1038/s41467-021-23469-2 PMC8160346

[ref55] Group AC-TfIwC-S (2022). Efficacy and safety of two neutralising monoclonal antibody therapies, sotrovimab and BRII-196 plus BRII-198, for adults hospitalised with COVID-19 (TICO): a randomised controlled trial. Lancet Infect. Dis. 22, 622–635. doi: 10.1016/S1473-3099(21)00751-9, PMID: 34953520 PMC8700279

[ref56] Guo Y. Huang L. Zhang G. Yao Y. Zhou H. Shen S.. (2021). A SARS-CoV-2 neutralizing antibody with extensive spike binding coverage and modified for optimal therapeutic outcomes. Nat. Commun. 12:2623. 33976198 10.1038/s41467-021-22926-2 PMC8113581

[ref57] Guo L. Wang G. Wang Y. Zhang Q. Ren L. Gu X.. (2022). SARS-CoV-2-specific antibody and T-cell responses 1 year after infection in people recovered from COVID-19: a longitudinal cohort study. Lancet Microbe 3, e348–e356. doi: 10.1016/S2666-5247(22)00036-2, PMID: 35345417 PMC8942480

[ref58] Haagmans B. L. Noack D. Okba N. M. A. Li W. Wang C. Bestebroer T.. (2021). SARS-CoV-2 neutralizing human antibodies protect against lower respiratory tract disease in a hamster model. J. Infect. Dis. 223, 2020–2028. doi: 10.1093/infdis/jiab289, PMID: 34043806 PMC8243397

[ref59] Hansen J. Baum A. Pascal K. E. Russo V. Giordano S. Wloga E.. (2020). Studies in humanized mice and convalescent humans yield a SARS-CoV-2 antibody cocktail. Science 369, 1010–1014. doi: 10.1126/science.abd0827, PMID: 32540901 PMC7299284

[ref60] Hasoksuz M. Kilic S. Sarac F. (2020). Coronaviruses and SARS-COV-2. Turk. J. Med. Sci. 50, 549–556. 10.3906/sag-2004-127 PMC7195990 32293832

[ref61] Hirzel C. Grandgirard D. Surial B. Wider M. F. Leppert D. Kuhle J.. (2022). Neuro-axonal injury in COVID-19: the role of systemic inflammation and SARS-CoV-2 specific immune response. Ther. Adv. Neurol. Disord. 15:17562864221080528. doi: 10.1177/17562864221080528, PMID: 35299779 PMC8922213

[ref62] Ho M. (2020). Perspectives on the development of neutralizing antibodies against SARS-CoV-2. Antib Ther. 3, 109–114. doi: 10.1093/abt/tbaa009, PMID: 32566896 PMC7291920

[ref63] Hoffmann M. Hofmann-Winkler H. Smith J. C. Kruger N. Arora P. Sorensen L. K.. (2021). Camostat mesylate inhibits SARS-CoV-2 activation by TMPRSS2-related proteases and its metabolite GBPA exerts antiviral activity. EBioMedicine 65:103255. doi: 10.1016/j.ebiom.2021.103255, PMID: 33676899 PMC7930809

[ref64] Hoffmann M. Kleine-Weber H. Schroeder S. Kruger N. Herrler T. Erichsen S.. (2020). SARS-CoV-2 cell entry depends on ACE2 and TMPRSS2 and is blocked by a clinically proven protease inhibitor. Cells 181, 271–280 e278. doi: 10.1016/j.cell.2020.02.052, PMID: 32142651 PMC7102627

[ref65] Hou X. Tian L. Zhou L. Jia X. Kong L. Xue Y.. (2021). Intravenous immunoglobulin-based adjuvant therapy for severe COVID-19: a single-center retrospective cohort study. Virol. J. 18:101. 34020680 10.1186/s12985-021-01575-3 PMC8139546

[ref66] Huang C. Fei L. Li W. Xu W. Xie X. Li Q.. (2021). Efficacy evaluation of intravenous immunoglobulin in non-severe patients with COVID-19: a retrospective cohort study based on propensity score matching. Int. J. Infect. Dis. 105, 525–531. doi: 10.1016/j.ijid.2021.01.009, PMID: 33434674 PMC7833031

[ref67] Huang S. Fishell G. (2022). In SARS-CoV-2, astrocytes are in it for the long haul. Proc. Natl. Acad. Sci. 119:e2209130119. doi: 10.1073/pnas.2209130119, PMID: 35858460 PMC9335203

[ref68] Iba T. Levy J. H. (2022). The roles of platelets in COVID-19-associated coagulopathy and vaccine-induced immune thrombotic thrombocytopenia. Trends Cardiovasc. Med. 32, 1–9. doi: 10.1016/j.tcm.2021.08.012, PMID: 34455073 PMC8390120

[ref69] Imai K. Tabata S. Ikeda M. Noguchi S. Kitagawa Y. Matuoka M.. (2020). Clinical evaluation of an immunochromatographic IgM/IgG antibody assay and chest computed tomography for the diagnosis of COVID-19. J. Clin. Virol. 128:104393. doi: 10.1016/j.jcv.2020.104393, PMID: 32387968 PMC7191278

[ref70] Jahrsdorfer B. Gross R. Seidel A. Wettstein L. Ludwig C. Schwarz T.. (2021). Characterization of the SARS-CoV-2 neutralization potential of COVID-19-convalescent donors. J. Immunol. 206, 2614–2622. doi: 10.4049/jimmunol.2100036, PMID: 33980583

[ref71] Jaimes J. A. Andre N. M. Chappie J. S. Millet J. K. Whittaker G. R. (2020). Phylogenetic analysis and structural modeling of SARS-CoV-2 spike protein reveals an evolutionary distinct and Proteolytically sensitive activation loop. J. Mol. Biol. 432, 3309–3325. doi: 10.1016/j.jmb.2020.04.009, PMID: 32320687 PMC7166309

[ref72] Jiang S. Zhang X. Yang Y. Hotez P. J. Du L. (2020). Neutralizing antibodies for the treatment of COVID-19. Nat. Biomed. Eng. 4, 1134–1139. doi: 10.1038/s41551-020-00660-2, PMID: 33293725 PMC7891858

[ref73] Jin D. Wei J. Sun J. (2021). Analysis of the molecular mechanism of SARS-CoV-2 antibodies. Biochem. Biophys. Res. Commun. 566, 45–52. doi: 10.1016/j.bbrc.2021.06.001, PMID: 34116356 PMC8179121

[ref74] Johnson A. M. Barigye R. Saminathan H. (2021). Perspectives on the use and risk of adverse events associated with cytokine-storm targeting antibodies and challenges associated with development of novel monoclonal antibodies for the treatment of COVID-19 clinical cases. Hum. Vaccin. Immunother. 17, 2824–2840. doi: 10.1080/21645515.2021.1908060, PMID: 33974497 PMC8127167

[ref75] Jovcevska I. Muyldermans S. (2020). The therapeutic potential of Nanobodies. BioDrugs 34, 11–26. doi: 10.1007/s40259-019-00392-z, PMID: 31686399 PMC6985073

[ref76] Ju B. Zhang Q. Ge J. Wang R. Sun J. Ge X.. (2020). Human neutralizing antibodies elicited by SARS-CoV-2 infection. Nature 584, 115–119. doi: 10.1038/s41586-020-2380-z, PMID: 32454513

[ref77] Kannan S. Shaik Syed Ali P. Sheeza A. (2021). Omicron (B.1.1.529) - variant of concern - molecular profile and epidemiology: a mini review. Eur. Rev. Med. Pharmacol. Sci. 25, 8019–8022. doi: 10.26355/eurrev_202112_27653, PMID: 34982466

[ref78] Kim C. Ryu D. K. Lee J. Kim Y. I. Seo J. M. Kim Y. G.. (2021). A therapeutic neutralizing antibody targeting receptor binding domain of SARS-CoV-2 spike protein. Nat. Commun. 12:288. 33436577 10.1038/s41467-020-20602-5 PMC7803729

[ref79] Klasse P. J. (2014). Neutralization of virus infectivity by antibodies: old problems in new perspectives. Adv. Biol. doi: 10.1155/2014/157895, PMID: 27099867 PMC4835181

[ref80] Klasse P. J. Moore J. P. (2020). Antibodies to SARS-CoV-2 and their potential for therapeutic passive immunization. elife 10.7554/eLife.57877 PMC7311167 32573433

[ref81] Kombe Kombe A. J. Zahid A. Mohammed A. Shi R. Jin T. (2021). Potent molecular feature-based neutralizing monoclonal antibodies as promising therapeutics against SARS-CoV-2 infection. Front. Mol. Biosci. 8:670815. doi: 10.3389/fmolb.2021.670815, PMID: 34136533 PMC8201996

[ref82] Korompoki E. Gavriatopoulou M. Hicklen R. S. Ntanasis-Stathopoulos I. Kastritis E. Fotiou D.. (2021). Epidemiology and organ specific sequelae of post-acute COVID19: a narrative review. J. Inf. 83, 1–16. doi: 10.1016/j.jinf.2021.05.004, PMID: 33992686 PMC8118709

[ref83] Kumari M. Lu R.-M. Li M.-C. Huang J.-L. Hsu F.-F. Ko S.-H.. (2022). A critical overview of current progress for COVID-19: development of vaccines, antiviral drugs, and therapeutic antibodies. J. Biomed. Sci. 29:68. 36096815 10.1186/s12929-022-00852-9 PMC9465653

[ref84] Lagunas-Rangel F. A. Chavez-Valencia V. (2021). What do we know about the antibody responses to SARS-CoV-2? Immunobiology 226:152054. doi: 10.1016/j.imbio.2021.152054, PMID: 33524881 PMC7826124

[ref85] Levi M. van Es N. (2022). COVID-19 associated coagulopathy and thrombosis in cancer. Thromb. Res. 213, S72–S76. doi: 10.1016/j.thromres.2021.12.006, PMID: 36210564 PMC9134033

[ref86] Levine M. M. (2019). Monoclonal antibody therapy for Ebola virus disease. N. Engl. J. Med. 381, 2365–2366. doi: 10.1056/NEJMe1915350, PMID: 31774948

[ref87] Li K. Meyerholz D. K. Bartlett J. A. McCray P. B. Jr. (2021). The TMPRSS2 inhibitor Nafamostat reduces SARS-CoV-2 pulmonary infection in mouse models of COVID-19. MBio 12:e0097021. doi: 10.1128/mBio.00970-21, PMID: 34340553 PMC8406266

[ref88] Liang H. Nian X. Wu J. Liu D. Feng L. Lu J.. (2022). COVID-19 vaccination boosts the potency and breadth of the immune response against SARS-CoV-2 among recovered patients in Wuhan. Cell Discov. 8:131. 36494338 10.1038/s41421-022-00496-x PMC9734167

[ref89] Liu L. Iketani S. Guo Y. Chan J. F.-W. Wang M. Liu L.. (2022). Striking antibody evasion manifested by the omicron variant of SARS-CoV-2. Nature 602, 676–681. doi: 10.1038/s41586-021-04388-0, PMID: 35016198

[ref90] Liu Y. Soh W. T. Kishikawa J. I. Hirose M. Nakayama E. E. Li S.. (2021). An infectivity-enhancing site on the SARS-CoV-2 spike protein targeted by antibodies. Cells 184, 3452–66 e18. doi: 10.1016/j.cell.2021.05.032, PMID: 34139176 PMC8142859

[ref91] Liu L. Wei Q. Lin Q. Fang J. Wang H. Kwok H.. (2019). Anti-spike IgG causes severe acute lung injury by skewing macrophage responses during acute SARS-CoV infection. JCI. Insight 4. doi: 10.1172/jci.insight.123158, PMID: 30830861 PMC6478436

[ref92] Lok S. M. (2021). An NTD supersite of attack. Cell Host Microbe 29, 744–746. doi: 10.1016/j.chom.2021.04.010, PMID: 33984277 PMC8114578

[ref93] Long Q. X. Liu B. Z. Deng H. J. Wu G. C. Deng K. Chen Y. K.. (2020). Antibody responses to SARS-CoV-2 in patients with COVID-19. Nat. Med. 26, 845–848. doi: 10.1038/s41591-020-0897-1, PMID: 32350462

[ref94] Mahalakshmi A. M. Ray B. Tuladhar S. Bhat A. Paneyala S. Patteswari D.. (2021). Does COVID-19 contribute to development of neurological disease? Immun. Inflam. Dis. 9, 48–58. doi: 10.1002/iid3.387, PMID: 33332737 PMC7860611

[ref95] Makdasi E. Levy Y. Alcalay R. Noy-Porat T. Zahavy E. Mechaly A.. (2021). Neutralizing monoclonal anti-SARS-CoV-2 antibodies isolated from immunized rabbits define novel vulnerable spike-protein epitope. Viruses 13. doi: 10.3390/v13040566, PMID: 33810465 PMC8065470

[ref96] Matveeva O. Nechipurenko Y. Lagutkin D. Yegorov Y. E. Kzhyshkowska J. (2022). SARS-CoV-2 infection of phagocytic immune cells and COVID-19 pathology: antibody-dependent as well as independent cell entry. Front. Immunol. 13:7128. 10.3389/fimmu.2022.1050478 PMC9751203 36532011

[ref97] McCoy J. Goren A. Cadegiani F. A. Vaño-Galván S. Kovacevic M. Situm M.. (2021). Proxalutamide reduces the rate of hospitalization for COVID-19 male outpatients: a randomized double-blinded placebo-controlled trial. Front. Med. 8:668698. doi: 10.3389/fmed.2021.668698, PMID: 34350193 PMC8326462

[ref98] Mehandru S. Merad M. (2022). Pathological sequelae of long-haul COVID. Nat. Immunol. 23, 194–202. doi: 10.1038/s41590-021-01104-y, PMID: 35105985 PMC9127978

[ref99] Meng B. Abdullahi A. Ferreira I. Goonawardane N. Saito A. Kimura I.. (2022). Altered TMPRSS2 usage by SARS-CoV-2 omicron impacts infectivity and fusogenicity. Nature 603, 706–714. doi: 10.1038/s41586-022-04474-x, PMID: 35104837 PMC8942856

[ref100] Min L. Sun Q. (2021). Antibodies and vaccines target RBD of SARS-CoV-2. Front. Mol. Biosci. 8:671633. doi: 10.3389/fmolb.2021.671633, PMID: 33968996 PMC8100443

[ref101] Mistry P. Barmania F. Mellet J. Peta K. Strydom A. Viljoen I. M.. (2022). SARS-CoV-2 variants, vaccines, and host immunity. Front. Immunol. 12:5400. 10.3389/fimmu.2021.809244 PMC8761766 35046961

[ref102] Mohapatra R. K. Tiwari R. Sarangi A. K. Islam M. R. Chakraborty C. Dhama K. (2022). Omicron (B.1.1.529) variant of SARS-CoV-2: concerns, challenges, and recent updates. J. Med. Virol. 94, 2336–2342. doi: 10.1002/jmv.27633, PMID: 35118666 PMC9015506

[ref103] Muyldermans S. (2013). Nanobodies: natural single-domain antibodies. Annu. Rev. Biochem. 82, 775–797. doi: 10.1146/annurev-biochem-063011-092449, PMID: 23495938

[ref104] Negi N. Maurya S. P. Singh R. Das B. K. (2022). An update on host immunity correlates and prospects of re-infection in COVID-19. Int. Rev. Immunol. 41, 367–392. doi: 10.1080/08830185.2021.2019727, PMID: 34961403 PMC8787841

[ref105] Nguyen N. N. Houhamdi L. Hoang V. T. Delerce J. Delorme L. Colson P.. (2022). SARS-CoV-2 reinfection and COVID-19 severity. Emerg. Microbes Infect. 11, 894–901. doi: 10.1080/22221751.2022.2052358, PMID: 35264078 PMC8942490

[ref106] Nickols N. G. Mi Z. DeMatt E. Biswas K. Clise C. E. Huggins J. T.. (2022). Effect of androgen suppression on clinical outcomes in hospitalized men with COVID-19: the HITCH randomized clinical trial. JAMA Netw. Open 5:e227852. doi: 10.1001/jamanetworkopen.2022.7852, PMID: 35438754 PMC9020208

[ref107] Ning Q. Wu D. Wang X. Xi D. Chen T. Chen G.. (2022). The mechanism underlying extrapulmonary complications of the coronavirus disease 2019 and its therapeutic implication. Signal Transduct. Target. Ther. 7, 1–33. 35197452 10.1038/s41392-022-00907-1 PMC8863906

[ref108] Okba N. M. A. Muller M. A. Li W. Wang C. GeurtsvanKessel C. H. Corman V. M.. (2020). Severe acute respiratory syndrome coronavirus 2-specific antibody responses in coronavirus disease patients. Emerg. Infect. Dis. 26, 1478–1488. doi: 10.3201/eid2607.200841, PMID: 32267220 PMC7323511

[ref109] Organization WH (2021). Evaluation of COVID-19 vaccine effectiveness: Interim guidance, 17 march 2021. World Health Organization.

[ref110] Pandey K. Acharya A. Mohan M. Ng C. L. Reid S. P. Byrareddy S. N. (2021). Animal models for SARS-CoV-2 research: a comprehensive literature review. Transbound. Emerg. Dis. 68, 1868–1885. doi: 10.1111/tbed.13907, PMID: 33128861 PMC8085186

[ref111] Pantaleo G. Correia B. Fenwick C. Joo V. S. Perez L. (2022). Antibodies to combat viral infections: development strategies and progress. Nat. Rev. Drug Discov. 21, 676–696. doi: 10.1038/s41573-022-00495-3, PMID: 35725925 PMC9207876

[ref112] Perricone C. Triggianese P. Bursi R. Cafaro G. Bartoloni E. Chimenti M. S.. (2021). Intravenous immunoglobulins at the crossroad of autoimmunity and viral infections. Microorganisms 9. doi: 10.3390/microorganisms9010121, PMID: 33430200 PMC7825648

[ref113] Perween R. Ahmed S. Shrivastava T. Parray H. A. Singh B. Pindari K. S.. (2021). A rapid novel strategy for screening of antibody phage libraries for production, purification, and functional characterization of amber stop codons containing single-chain antibody fragments. Biotechnol. Prog. 37:e3136. doi: 10.1002/btpr.3136, PMID: 33620776

[ref114] Piccoli L. Park Y. J. Tortorici M. A. Czudnochowski N. Walls A. C. Beltramello M.. (2020). Mapping neutralizing and Immunodominant sites on the SARS-CoV-2 spike receptor-binding domain by structure-guided high-resolution serology. Cells 183, 1024–42 e21. doi: 10.1016/j.cell.2020.09.037, PMID: 32991844 PMC7494283

[ref115] Pilz S. Theiler-Schwetz V. Trummer C. Krause R. Ioannidis J. P. (2022). SARS-CoV-2 reinfections: overview of efficacy and duration of natural and hybrid immunity. Environ. Res. 209:112911. doi: 10.1016/j.envres.2022.112911, PMID: 35149106 PMC8824301

[ref116] Pisil Y. Yazici Z. Shida H. Miura T. (2021). Is SARS-CoV-2 neutralized more effectively by IgM and IgA than IgG having the same fab region? Pathogens 10. doi: 10.3390/pathogens10060751, PMID: 34199224 PMC8231813

[ref117] Pizzato M. Baraldi C. Boscato Sopetto G. Finozzi D. Gentile C. Gentile M. D.. (2022). SARS-CoV-2 and the host cell: a tale of interactions. Front. Virol. 1:815388. doi: 10.3389/fviro.2021.815388

[ref118] Ramasamy M. N. Minassian A. M. Ewer K. J. Flaxman A. L. Folegatti P. M. Owens D. R.. (2021). Safety and immunogenicity of ChAdOx1 nCoV-19 vaccine administered in a prime-boost regimen in young and old adults (COV002): a single-blind, randomised, controlled, phase 2/3 trial. Lancet 396, 1979–1993. doi: 10.1016/S0140-6736(20)32466-1, PMID: 33220855 PMC7674972

[ref119] Rank A. Tzortzini A. Kling E. Schmid C. Claus R. Löll E.. (2021). One year after mild COVID-19: the majority of patients maintain specific immunity, but one in four still suffer from long-term symptoms. J. Clin. Med. 10:3305. doi: 10.3390/jcm10153305, PMID: 34362088 PMC8347559

[ref120] Razonable R. R. Chen P. (2022). Editorial: neutralizing antibodies in the prevention and treatment of COVID-19. Front. Immunol. 13:938069. doi: 10.3389/fimmu.2022.938069, PMID: 35720338 PMC9198713

[ref121] Rogers T. F. Zhao F. Huang D. Beutler N. Burns A. He W. T.. (2020). Isolation of potent SARS-CoV-2 neutralizing antibodies and protection from disease in a small animal model. Science 369, 956–963. doi: 10.1126/science.abc7520, PMID: 32540903 PMC7299280

[ref122] Rothenburg J. Rink-Baron S. Mueller L. Ostermann P. N. Fischer J. Stegbauer J.. (2021). COVID-19 antibody donation using immunoadsorption: report of two cases. Transfus. Apher. Sci. 60:103193. doi: 10.1016/j.transci.2021.103193, PMID: 34147358 PMC8205282

[ref123] Ruiz-Galiana J. Ramos P. D. L. García-Botella A. García-Lledó A. Gómez-Pavón J. del Castillo J. G.. (2022). Persistence and viability of SARS-CoV-2 in primary infection and reinfections. Rev. Esp. Quimioter. 35:1. doi: 10.37201/req/129.2021, PMID: 34661382 PMC8790642

[ref124] Sanna V. Satta S. Hsiai T. Sechi M. (2022). Development of targeted nanoparticles loaded with antiviral drugs for SARS-CoV-2 inhibition. Eur. J. Med. Chem. 231:114121. doi: 10.1016/j.ejmech.2022.114121, PMID: 35114539 PMC8755562

[ref125] Shi R. Shan C. Duan X. Chen Z. Liu P. Song J.. (2020). A human neutralizing antibody targets the receptor-binding site of SARS-CoV-2. Nature 584, 120–124. doi: 10.1038/s41586-020-2381-y, PMID: 32454512

[ref126] Shi J. Wen Z. Zhong G. Yang H. Wang C. Huang B.. (2020). Susceptibility of ferrets, cats, dogs, and other domesticated animals to SARS-coronavirus 2. Science 368, 1016–1020. doi: 10.1126/science.abb7015, PMID: 32269068 PMC7164390

[ref127] Shimohata T. (2022). Neuro-COVID-19. Clin. Exp. Neuroimmunol. 13, 17–23. doi: 10.1111/cen3.12676, PMID: 34899999 PMC8652810

[ref128] Shirbhate E. Pandey J. Patel V. K. Kamal M. Jawaid T. Gorain B.. (2021). Understanding the role of ACE-2 receptor in pathogenesis of COVID-19 disease: a potential approach for therapeutic intervention. Pharmacol. Rep. 73, 1539–1550. doi: 10.1007/s43440-021-00303-6, PMID: 34176080 PMC8236094

[ref129] Shrwani K. Sharma R. Krishnan M. Jones T. Mayora-Neto M. Cantoni D.. (2021). Detection of serum cross-reactive antibodies and memory response to SARS-CoV-2 in Prepandemic and post-COVID-19 convalescent samples. J. Infect. Dis. 224, 1305–1315. doi: 10.1093/infdis/jiab333, PMID: 34161567 PMC8557674

[ref130] Sia S. F. Yan L. M. Chin A. W. H. Fung K. Choy K. T. Wong A. Y. L.. (2020). Pathogenesis and transmission of SARS-CoV-2 in golden hamsters. Nature 583, 834–838. doi: 10.1038/s41586-020-2342-5, PMID: 32408338 PMC7394720

[ref131] Silva-Beltrán N. P. Balderrama-Carmona A. P. Gálvez-Ruíz J.-C. Umsza-Guez M. A. Bustos E. R. (2022). “Biotechnological strategies in the intervention and treatment of COVID-19” in Frontiers of COVID-19: Scientific and clinical aspects of the novel coronavirus 2019 (Springer), 421–442.

[ref132] Song D. Wang W. Dong C. Ning Z. Liu X. Liu C.. (2021). Structure and function analysis of a potent human neutralizing antibody CA521(FALA) against SARS-CoV-2. Commun. Biol. 4:500. 33893388 10.1038/s42003-021-02029-w PMC8065039

[ref133] Steinman J. B. Lum F. M. Ho P. P.-K. Kaminski N. Steinman L. (2020). Reduced development of COVID-19 in children reveals molecular checkpoints gating pathogenesis illuminating potential therapeutics. Proc. Natl. Acad. Sci. 117, 24620–24626. doi: 10.1073/pnas.2012358117, PMID: 32883878 PMC7547272

[ref134] Sterlin D. Mathian A. Miyara M. Mohr A. Anna F. Claer L.. (2021). IgA dominates the early neutralizing antibody response to SARS-CoV-2. Sci. Transl. Med. 13. doi: 10.1126/scitranslmed.abd2223, PMID: 33288662 PMC7857408

[ref135] Suvarnapathaki S. Chauhan D. Nguyen A. Ramalingam M. Camci-Unal G. (2022). Advances in targeting ACE2 for developing COVID-19 therapeutics. Ann. Biomed. Eng., 1–16. 10.1007/s10439-022-03094-w PMC9581451 36261668

[ref136] Tabll A. A. Shahein Y. E. Omran M. M. Elnakib M. M. Ragheb A. A. Amer K. E. (2021). A review of monoclonal antibodies in COVID-19: role in immunotherapy, vaccine development and viral detection. Hum. Antibodies 29, 179–191. doi: 10.3233/HAB-200441, PMID: 33998533

[ref137] Tai W. Zhang X. He Y. Jiang S. Du L. (2020). Identification of SARS-CoV RBD-targeting monoclonal antibodies with cross-reactive or neutralizing activity against SARS-CoV-2. Antivir. Res. 179:104820. doi: 10.1016/j.antiviral.2020.104820, PMID: 32405117 PMC7219369

[ref138] Tay M. Z. Poh C. M. Rénia L. MacAry P. A. Ng L. F. (2020). The trinity of COVID-19: immunity, inflammation and intervention. Nat. Rev. Immunol. 20, 363–374. doi: 10.1038/s41577-020-0311-8, PMID: 32346093 PMC7187672

[ref139] Terpos E. Stellas D. Rosati M. Sergentanis T. N. Hu X. Politou M.. (2021). SARS-CoV-2 antibody kinetics eight months from COVID-19 onset: persistence of spike antibodies but loss of neutralizing antibodies in 24% of convalescent plasma donors. Eur. J. Intern. Med. 89, 87–96. doi: 10.1016/j.ejim.2021.05.010, PMID: 34053848 PMC8128693

[ref140] Tian X. Li C. Huang A. Xia S. Lu S. Shi Z.. (2020). Potent binding of 2019 novel coronavirus spike protein by a SARS coronavirus-specific human monoclonal antibody. Emerg. Microbes Infect. 9, 382–385. doi: 10.1080/22221751.2020.1729069, PMID: 32065055 PMC7048180

[ref141] Vaisman-Mentesh A. Gutierrez-Gonzalez M. DeKosky B. J. Wine Y. (2020). The molecular mechanisms that underlie the immune biology of anti-drug antibody formation following treatment with monoclonal antibodies. Front. Immunol. 11:1951. 33013848 10.3389/fimmu.2020.01951 PMC7461797

[ref142] Van Rompay K. K. A. Olstad K. J. Sammak R. L. Dutra J. Watanabe J. K. Usachenko J. L.. (2021). Early treatment with a combination of two potent neutralizing antibodies improves clinical outcomes and reduces virus replication and lung inflammation in SARS-CoV-2 infected macaques. PLoS Pathog. 17:e1009688. doi: 10.1371/journal.ppat.1009688, PMID: 34228761 PMC8284825

[ref143] VanBlargan L. A. Errico J. M. Halfmann P. J. Zost S. J. Crowe J. E. Jr. Purcell L. A.. (2022). An infectious SARS-CoV-2 B.1.1.529 omicron virus escapes neutralization by therapeutic monoclonal antibodies. Nat. Med. 28, 490–495. doi: 10.1038/s41591-021-01678-y, PMID: 35046573 PMC8767531

[ref144] Veenhuis R. T. Zeiss C. J. (2021). Animal models of COVID-19 II. Comparative immunology. ILAR J. 62, 17–34. doi: 10.1093/ilar/ilab010, PMID: 33914873 PMC8135340

[ref145] Verkhivker G. M. Di Paola L. (2021). Integrated biophysical modeling of the SARS-CoV-2 spike protein binding and allosteric interactions with antibodies. J. Phys. Chem. B 125, 4596–4619. doi: 10.1021/acs.jpcb.1c00395, PMID: 33929853

[ref146] Verkhratsky A. Li Q. Melino S. Melino G. Shi Y. (2020). Can COVID-19 pandemic boost the epidemic of neurodegenerative diseases? Biol. Direct 15, 1–8. 33246479 10.1186/s13062-020-00282-3 PMC7691955

[ref147] von Rhein C. Scholz T. Henss L. Kronstein-Wiedemann R. Schwarz T. Rodionov R. N.. (2021). Comparison of potency assays to assess SARS-CoV-2 neutralizing antibody capacity in COVID-19 convalescent plasma. J. Virol. Methods 288:114031. doi: 10.1016/j.jviromet.2020.114031, PMID: 33275926 PMC7707675

[ref148] Voss J. E. (2021). Engineered single-domain antibodies tackle COVID variants. Nature 595, 176–178. doi: 10.1038/d41586-021-01721-5, PMID: 34193999

[ref149] Walls A. C. Park Y. J. Tortorici M. A. Wall A. McGuire A. T. Veesler D. (2020). Structure, function, and antigenicity of the SARS-CoV-2 spike glycoprotein. Cells 181, 281–92 e6. doi: 10.1016/j.cell.2020.02.058, PMID: 32155444 PMC7102599

[ref150] Walls A. C. Xiong X. Park Y. J. Tortorici M. A. Snijder J. Quispe J.. (2019). Unexpected receptor functional mimicry elucidates activation of coronavirus fusion. Cells 176, 1026–39 e15. doi: 10.1016/j.cell.2018.12.028, PMID: 30712865 PMC6751136

[ref151] Wang S. Peng Y. Wang R. Jiao S. Wang M. Huang W.. (2020). Characterization of neutralizing antibody with prophylactic and therapeutic efficacy against SARS-CoV-2 in rhesus monkeys. Nat. Commun. 11:5752. 33188207 10.1038/s41467-020-19568-1 PMC7666115

[ref152] Wec A. Z. Wrapp D. Herbert A. S. Maurer D. P. Haslwanter D. Sakharkar M.. (2020). Broad neutralization of SARS-related viruses by human monoclonal antibodies. Science 369, 731–736. doi: 10.1126/science.abc7424, PMID: 32540900 PMC7299279

[ref153] Weinreich D. M. Sivapalasingam S. Norton T. Ali S. Gao H. Bhore R.. (2021). REGN-COV2, a neutralizing antibody cocktail, in outpatients with Covid-19. N. Engl. J. Med. 384, 238–251. doi: 10.1056/NEJMoa2035002, PMID: 33332778 PMC7781102

[ref154] Wendel S. Land K. Devine D. V. Daly J. Bazin R. Tiberghien P.. (2021). Lessons learned in the collection of convalescent plasma during the COVID-19 pandemic. Vox Sang. 116, 872–879. doi: 10.1111/vox.13096, PMID: 33772791 PMC8250874

[ref155] Willyard C. (2022). What the omicron wave is revealing about human immunity. Nature 602, 22–25. doi: 10.1038/d41586-022-00214-3, PMID: 35110764

[ref156] Xiang H. R. Cheng X. Li Y. Luo W. W. Zhang Q. Z. Peng W. X. (2021). Efficacy of IVIG (intravenous immunoglobulin) for corona virus disease 2019 (COVID-19): a meta-analysis. Int. Immunopharmacol. 96:107732. doi: 10.1016/j.intimp.2021.107732, PMID: 34162133 PMC8084608

[ref157] Xiong H. L. Wu Y. T. Cao J. L. Yang R. Liu Y. X. Ma J.. (2020). Robust neutralization assay based on SARS-CoV-2 S-protein-bearing vesicular stomatitis virus (VSV) pseudovirus and ACE2-overexpressing BHK21 cells. Emerg. Microbes Infect. 9, 2105–2113. doi: 10.1080/22221751.2020.1815589, PMID: 32893735 PMC7534347

[ref158] Xu J. Xu K. Jung S. Conte A. Lieberman J. Muecksch F.. (2021). Nanobodies from camelid mice and llamas neutralize SARS-CoV-2 variants. Nature 595, 278–282. doi: 10.1038/s41586-021-03676-z, PMID: 34098567 PMC8260353

[ref159] Xu J. Zhao S. Teng T. Abdalla A. E. Zhu W. Xie L.. (2020). Systematic comparison of two animal-to-human transmitted human coronaviruses: SARS-CoV-2 and SARS-CoV. Viruses 12. doi: 10.3390/v12020244, PMID: 32098422 PMC7077191

[ref160] Xue X. Fan X. Qu Q. Wu G. (2016). Bioscreening and expression of a camel anti-CTGF VHH nanobody and its renaturation by a novel dialysis-dilution method. AMB Express 6:72. 27620736 10.1186/s13568-016-0249-1 PMC5019992

[ref161] Yang S. Cao L. Xu W. Xu T. Zheng B. Ji Y.. (2022). Comparison of model-specific histopathology in mouse models of COVID-19. J. Med. Virol. 94, 3605–3612. doi: 10.1002/jmv.27747, PMID: 35355296 PMC9088385

[ref162] Yang X.-h. Deng W. Tong Z. Liu Y.-x. Zhang L.-f. Zhu H.. (2007). Mice transgenic for human angiotensin-converting enzyme 2 provide a model for SARS coronavirus infection. Comp. Med. 57, 450–459. PMID: 17974127

[ref163] Yokoyama A. P. H. Wendel S. Bonet-Bub C. Fachini R. M. Dametto A. P. F. Blumm F.. (2021). COVID-19 convalescent plasma cohort study: evaluation of the association between both donor and recipient neutralizing antibody titers and patient outcomes. Transfusion 61, 2295–2306. doi: 10.1111/trf.16573, PMID: 34173248 PMC8447313

[ref164] Yonemura S. Hartson L. Dutt T. S. Henao-Tamayo M. Goodrich R. Marschner S. (2021). Preservation of neutralizing antibody function in COVID-19 convalescent plasma treated using a riboflavin and ultraviolet light-based pathogen reduction technology. Vox Sang. 116, 1076–1083. doi: 10.1111/vox.13108, PMID: 33835489 PMC8251479

[ref165] Zhang J. Wu Q. Liu Z. Wang Q. Wu J. Hu Y.. (2021). Spike-specific circulating T follicular helper cell and cross-neutralizing antibody responses in COVID-19-convalescent individuals. Nat. Microbiol. 6, 51–58. doi: 10.1038/s41564-020-00824-5, PMID: 33199863

[ref166] Zhao H. Lu L. Peng Z. Chen L. L. Meng X. Zhang C.. (2022). SARS-CoV-2 omicron variant shows less efficient replication and fusion activity when compared with Delta variant in TMPRSS2-expressed cells. Emerg. Microbes Infect. 11, 277–283. doi: 10.1080/22221751.2021.2023329, PMID: 34951565 PMC8774049

[ref167] Zheng J. Deng Y. Zhao Z. Mao B. Lu M. Lin Y.. (2022). Characterization of SARS-CoV-2-specific humoral immunity and its potential applications and therapeutic prospects. Cell. Mol. Immunol. 19, 150–157. doi: 10.1038/s41423-021-00774-w, PMID: 34645940 PMC8513558

[ref168] Zhou D. Duyvesteyn H. M. E. Chen C. P. Huang C. G. Chen T. H. Shih S. R.. (2020). Structural basis for the neutralization of SARS-CoV-2 by an antibody from a convalescent patient. Nat. Struct. Mol. Biol. 27, 950–958. doi: 10.1038/s41594-020-0480-y, PMID: 32737466

[ref169] Zhou X. Ma F. Xie J. Yuan M. Li Y. Shaabani N.. (2021). Diverse immunoglobulin gene usage and convergent epitope targeting in neutralizing antibody responses to SARS-CoV-2. Cell Rep. 35:109109. doi: 10.1016/j.celrep.2021.109109, PMID: 33932326 PMC8064889

[ref170] Zhou P. Yang X. L. Wang X. G. Hu B. Zhang L. Zhang W.. (2020). A pneumonia outbreak associated with a new coronavirus of probable bat origin. Nature 579, 270–273. doi: 10.1038/s41586-020-2012-7, PMID: 32015507 PMC7095418

[ref171] Zolfaghari M. A. Ghadiri Moghaddam F. Rajput S. Karimi A. Naghi Vishteh M. Mahmoodpoor A.. (2022). SARS-CoV-2 vaccines: a double-edged sword throughout rapid evolution of COVID-19. Cell Biol. Int. 46, 2009–2017. doi: 10.1002/cbin.11903, PMID: 36047303 PMC9539123

